# Rapid whole genome sequencing impacts care and resource utilization in infants with congenital heart disease

**DOI:** 10.1038/s41525-021-00192-x

**Published:** 2021-04-22

**Authors:** Nathaly M. Sweeney, Shareef A. Nahas, Shimul Chowdhury, Sergey Batalov, Michelle Clark, Sara Caylor, Julie Cakici, John J. Nigro, Yan Ding, Narayanan Veeraraghavan, Charlotte Hobbs, David Dimmock, Stephen F. Kingsmore

**Affiliations:** 1 Rady Children’s Institute for Genomic Medicine, San Diego, CA USA; 2 Rady Children’s Hospital, San Diego, CA USA; 3 Department of Pediatrics, University of California San Diego, La Jolla, CA USA; 4 Department of Family Medicine and Public Health, University of California San Diego, La Jolla, CA USA; 5 Department of Surgery, University of California San Diego, La Jolla, CA USA

**Keywords:** Health care economics, Medical genomics, Congenital heart defects

## Abstract

Congenital heart disease (CHD) is the most common congenital anomaly and a major cause of infant morbidity and mortality. While morbidity and mortality are highest in infants with underlying genetic conditions, molecular diagnoses are ascertained in only ~20% of cases using widely adopted genetic tests. Furthermore, cost of care for children and adults with CHD has increased dramatically. Rapid whole genome sequencing (rWGS) of newborns in intensive care units with suspected genetic diseases has been associated with increased rate of diagnosis and a net reduction in cost of care. In this study, we explored whether the clinical utility of rWGS extends to critically ill infants with structural CHD through a retrospective review of rWGS study data obtained from inpatient infants < 1 year with structural CHD at a regional children’s hospital. rWGS diagnosed genetic disease in 46% of the enrolled infants. Moreover, genetic disease was identified five times more frequently with rWGS than microarray ± gene panel testing in 21 of these infants (rWGS diagnosed 43% versus 10% with microarray ± gene panels, *p* = 0.02). Molecular diagnoses ranged from syndromes affecting multiple organ systems to disorders limited to the cardiovascular system. The average daily hospital spending was lower in the time period post blood collection for rWGS compared to prior (*p* = 0.003) and further decreased after rWGS results (*p* = 0.000). The cost was not prohibitive to rWGS implementation in the care of this cohort of infants. rWGS provided timely actionable information that impacted care and there was evidence of decreased hospital spending around rWGS implementation.

## Introduction

Congenital heart disease (CHD) is the most common congenital anomaly and a major cause of infant morbidity and mortality^1,2^. Although surgical and medical advances have improved childhood survival in CHD from <20% in 1950 to >90% today, the incidence of the disease has remained unchanged over the last 3 decades, indicating little improvement in our understanding of the etiology of CHD^1–3^. Mortality has also remained disproportionately higher in infants and at lower sociodemographic indices indicating a persistent health disparity^2^. Furthermore, some survivors experience lifelong morbidity including developmental disabilities and report a suboptimal quality of life^4,5^.

A little studied determinant of outcomes surrounding surgical repair or medical management of CHD is the underlying etiology of disease. Routine clinical genetic testing, such as chromosomal microarray (CMA), reveals a genetic etiology in ~20% of symptomatic children with CHD and additional anomalies^6–8^. Genetic diseases are diagnosed much less commonly by routine clinical genetic tests in those with isolated CHD^6–8^. Genetic diseases complicate the management of CHD since they often affect other organ systems and may have profound consequences both for surgical and medical management^9^.

Rapid whole genome sequencing (rWGS) has recently become feasible for timely diagnosis of genetic diseases presenting at birth. Unlike other genetic tests, rWGS examines over 90% of the human genome for single-nucleotide changes, small insertions and deletions, and copy number variants^10^. rWGS examines the cause of thousands of genetic diseases, precluding the need for their prior consideration in the differential diagnosis. By identifying an underlying genetic etiology of presentations at birth, rWGS can enable individual tailoring of management, including prognostic determination and screening for complications. In a prospective study, WGS increased the rate of childhood genetic disease diagnosis fourfold compared with CMA alone and twofold compared with CMA and targeted gene sequencing^11^. Improvements in outcome of critically ill infants through rapid genomic sequencing have been well documented^12–14^.

In addition, rWGS of newborns in intensive care units with suspected genetic diseases has been shown to be associated with a net reduction in cost of care^14^. Cost of care for children and adults with CHD has increased dramatically—from $2.7 billion billed in 2002 to $7 billion in 2012 for children with CHD. For adult CHD, the amount billed increased from $543 million to $1.5 billion in the same time period^15^. In this retrospective review, we explored whether the clinical utility of rWGS extends to critically ill infants with structural CHD.

## Results

### Demographic and characteristics of enrolled probands

**Fig. 1:**
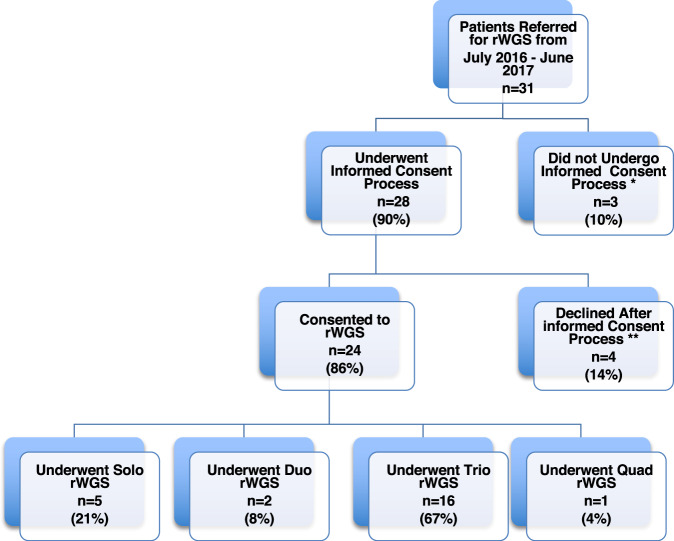
Flow diagram of families referred for rWGS and the rate of consent to the study. Eighty-six percent of families who underwent the informed consent process enrolled in the rWGS study. *Three families did not undergo informed consent process (one maxed out on number of contact attempts per research protocol, two for unknown reasons). The majority of families underwent trio rWGS (67%). **Four families declined participation after informed consent process: two secondary to concerns for lack of protection under GINA (Genetic Information Nondiscrimination Act of 2008, Pub. L. 110–233, 122 Stat. 881), one wanted more than just phenotype driven genetic information, and one did not follow up after consent process.

Thirty-one infants with structural CHD and their parents were referred for rWGS by the inpatient clinical team during an 11-month period (Fig. 1, Supplementary Table 5). Overall, 28 families underwent the informed consent process and 24 of them (86%) consented to participate in rWGS research, while 4 (14%) declined participation. Families were encouraged to participate as parent–infant trios. Overall, 16 of the 24 families (67%) received trio sequencing (proband and parents), 5 (21%) solo (proband only), 2 (8%) duo (proband and mother), and 1 (4%) quad (proband, parents and an affected sibling) (Fig. 1, Supplementary Table 5). All but one had only one affected family member (the proband). The family that underwent quad rWGS had two affected children and one affected parent. The etiology of CHD was not known in this family. Overall, 21 of 24 probands had clinical genetic testing in the form of microarray and/or targeted gene panels. Three children had no additional genetic testing (Supplementary Table 5). Limited findings on six patients included in the current manuscript were previously published in *npj Genomic Medicine* in 2018^14^.

Sixty-seven percent of the patients who underwent rWGS were male (Table 1). Families predominantly identified enrolled infants as Hispanic/Latino (42%). Twenty infants (83%) were enrolled from the neonatal intensive care unit, three (13%) from the cardiovascular intensive care unit and one (4%) from the inpatient gastroenterology unit. Eleven of the probands (45%) were premature (<37 weeks’ gestation) and ten (42%) had a birth weight less than 2.5 kg. Newborns were admitted to Rady Children’s Hospital either from home or transferred from birthing hospitals. All probands had symptom onset in the neonatal period.

**Table 1:** Demographic and clinical characteristics of the probands.

			Rapid whole genome sequencing			
			Total (*n* = 24)	Diagnostic (*n* = 11)	Negative (*n* = 13)	*p* value
Sex	Male		16 (67%)	9 (82%)	7 (54%)	0.21
Race and ethnicity	Caucasian		6 (25%)	0 (0%)	6 (46%)	0.02*
	Hispanic/Latino		10 (42%)	7 (64%)	3 (23%)	0.10
	African/African American		3 (12%)	1 (9%)	2 (15%)	1
	Asian/Native American/Pacific Islander		2 (8%)	0 (0%)	2 (15%)	0.49
	Other		3 (12%)	3 (27%)	0 (0%)	0.08
Source of nomination**	Level IV neonatal intensive care unit		20 (83%)	9 (82%)	11 (85%)	1
	Cardiovascular intensive care unit		3 (13%)	1 (9%)	2 (15%)	1
	Inpatient gastroenterology		1 (4%)	1 (9%)	0 (0%)	0.46
Birth characteristics	Gestational age	<37 weeks	11 (45%)	5 (42%)	6 (46%)	1
	Birth weight	<2.5 kg	10 (42%)	3 (27%)	7 (54%)	0.24
		Not recorded	1 (4%)	0 (0%)	1 (8%)	1
	Symptom onset	<1 month	24 (100%)	11 (100%)	13 (100%)	1
Additional systems involved	Musculoskeletal^a^		14 (58%)	6 (55%)	8 (62%)	1
	Genitourinary		11 (45%)	3 (27%)	8 (62%)	0.12
	Ear, nose, and throat		9 (38%)	4 (36%)	5 (38%)	1
	Neurological		6 (25%)	1 (9%)	5 (38%)	0.17
	Gastrointestinal/Hepatic		4 (17%)	1 (9%)	3 (23%)	0.60
	Hematological		4 (17%)	2 (18%)	2 (15%)	1
	Endocrine/Biochemical		2 (8%)	2 (18%)	0 (0%)	0.20
	Pulmonary		2 (8%)	1 (9%)	1 (8%)	1
	Ophthalmologic		2 (8%)	1 (9%)	1 (8%)	1
	Immunological		1 (4%)	0 (0%)	1 (8%)	1
Medical management	Inotropic support		20 (83%)	7 (64%)	13 (100%)	0.03**
	Respiratory support		23 (96%)	10 (91%)	13 (100%)	0.46
	Intubated		22 (92%)	9 (82%)	13 (100%)	0.20
	Antimicrobial treatment		21 (88%)	9 (82%)	12 (92%)	0.58
	≥5 subspecialist consults		17 (71%)	9 (82%)	8 (62%)	0.39
	Pretesting clinical genetics consultation		13 (54%)	7 (64%)	6 (46%)	0.39
Mortality			6 (25%)	1 (9%)	5 (38%)	0.17

Values shown are number (percentage) of subjects, except as indicated.

*Rate of diagnosis was significantly lower in Caucasian infants (*p* = 0.02).

**More infants in the nondiagnostic group required inotropic support at some time during their current hospitalization compared to infants in the diagnostic group (*p* = 0.03). *p* values for categorical variables were calculated using Fisher’s exact test.

^a^Includes arthrogryposis.

Besides CHD, additional organ system involvement was identified in some patients during the hospitalization: musculoskeletal in 14 (58%), genitourinary in 11 (45%), ear, nose, and throat in 9 (38%), and central nervous in 6 (25%). The patients were critically ill as evidenced by the use of inotropic support in 20 (83%) and respiratory support in 23 (96%), of whom 22 (92%) were intubated and mechanically ventilated. A total of 21 infants (88%) received antimicrobial treatment for suspected sepsis, and 17 (71%) had five or more subspecialist consults (Table 1). A significant difference was seen in inotropic use between the groups, which was higher in the undiagnosed group (100%) compared to the diagnostic group (64%, *p* = 0.03, difference 36%, 95% CI 5%, 65%) (Table 1). More than half (54%, 13/24) of the patients had a clinical genetic consultation prior to any genetic testing (Table 1).

### Rate of genetic diagnosis with rWGS, CMA, and gene panels

**Fig. 2:**
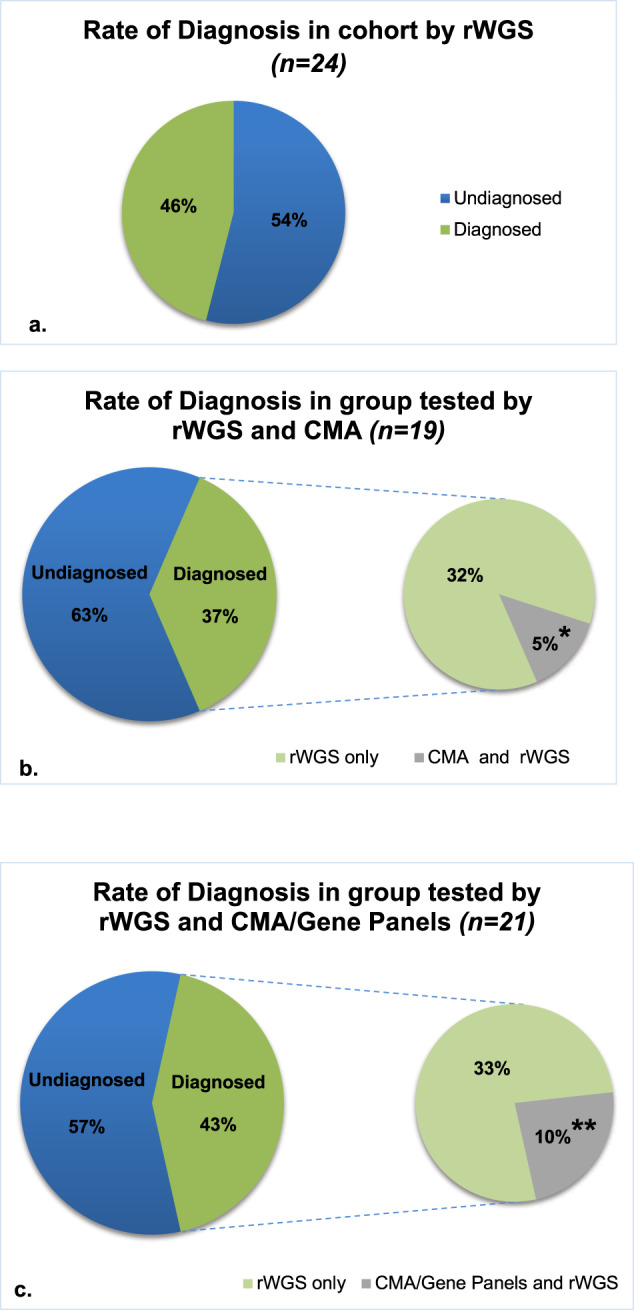
Rate of genetic diagnosis with rWGS, CMA and gene panels. **a**–**c** Rate of Genetic diagnosis with rWGS, CMA and gene panels. **a** Rate of diagnosis in cohort by rWGS. rWGS had a higher rate of diagnosis (11/24) in the cohort compared to microarray (1/19) and microarray +/− gene panel (2/21). **b** Rate of diagnosis in group tested by rWGS and CMA. The rate of diagnosis was statistically significant higher with rWGS compared to microarray (*p* = 0.04*; McNemar’s Test) when comparing the rate of diagnosis within the group that had both microarray and rWGS testing (*n* = 19). **c** Rate of diagnosis in group tested by rWGS and CMA/Gene Panels. When comparing the rate of diagnosis of rWGS within the group that received microarray +/− gene panels testing (*n* = 21) and rWGS, rWGS still outperformed in yielding a diagnosis (*p* = 0.02**; McNemar’s Test).

In assessing the rate of diagnosis the patients functioned as their own control, since they underwent both rWGS and had clinical genetic testing as ordered by the primary medical team. Sixty-four percent of the patients in the diagnostic group had a clinical genetic consultation. Based on these evaluations WES was recommended for one patient, CMA for three and gene panel testing for two. In the undiagnosed group, WGS sequencing was recommended for one and CMA for five. Overall, the rate of rWGS diagnosis was higher than that of CMA and/or targeted gene panels (Fig. 2a). Overall, 11 of the 24 probands (46%) obtained a molecular diagnosis of a genetic disease by rWGS, compared with two (10%) identified by clinical genetic testing. For the 19 patients who received both rWGS and CMA testing, the rate of diagnosis was statistically significant higher in the rWGS group (7 of 19 (37%)) compared to CMA group (1 of 19 (5%), *p* = 0.04, difference 32%, 95% CI 11%, 52%) (Fig. 2b). The rate of diagnosis by clinical genetic testing when combining CMA and targeted gene panel results, increased to 10% (2 of 21) and remained statistically significantly lower than the rate of diagnosis by rWGS (9 of 21 (43%), *p* = 0.02, difference 33%, 95% CI 13%, 54%) (Fig. 2c). The two diagnoses made by CMA or targeted gene panels were also made by rWGS. The rate of diagnosis was not significantly different between male (56%) and female probands (25%) (*p* = 0.21, difference 31%, 95% CI −10%, 59%). The overall mortality rate in the cohort was 25% (*n* = 6). Although mortality was higher in the undiagnosed group, *n* = 5 (38%), than in the diagnostic group, *n* = 1 (9%), the difference did not reach statistical significance (Table 1). The rate of diagnosis was significantly lower in Caucasian infants, *p* = 0.02, difference 46%, 95% CI −75%, −14%).

### Genetic diagnosis and impact on medical care

A total of 12 genetic diseases were identified in 11 patients (Table 2). The majority of diagnoses were associated with variants that occurred de novo (7 of 12, 58%), while 4 were inherited. Inheritance was autosomal dominant (AD) in the majority of diagnoses (10 of 12, 83%), while one was autosomal recessive (AR) and one X-linked dominant (XLD). Most diagnoses explained the cardiac and associated anomalies of the probands (9 of 11, 82%). Two infants (proband 18 and 24) received partial diagnoses—genetic diseases in which literature review failed to disclose a previous association with their type of CHD. The partial diagnoses did, however, explain the other organ system involvement, and informed medical management in both cases.

**Table 2:** Genetic diagnoses and effect on management.

Family ID^a^	rWGS	Gene(s)	Inheritance pattern(s)	De novo or inherited	Position dbSNP	Gene (c.) Coordinate(s)	Variant protein coordinate(s)	Diagnosis	Effect on management
12	Solo	ARID1B	AD	De novo	chr6:157495210	c.3096_3100delCAAAG	p.Lys1033ArgfsTer32	Coffin–Siris syndrome (OMIM# 135900)	Palliative care
18	Trio	POLR1C	AR	Inherited (maternal)	chr6:43487171	c.242T>C	p.Leu81Pro	Leukodystrophy, hypomyelinating (OMIM# 616494)	Enlistment of additional subspecialist
				Inherited (paternal)	chr6:43487520	c.326G>A	p.Arg109His		
20	Solo	TPM1	AD	N.d. (Duo)	chr15:63353108	c.533G>A	p.Arg178His	Left ventricular noncompaction syndrome (OMIM# 611878)	Listing for cardiac transplantation
24	Trio	PHEX	XLD	Inherited	chrX:22208578	c.1604C>T	p.Thr535Met	X-linked hypophosphatemic rickets syndrome (OMIM# 307800)	Enlistment of additional subspecialist
26	Trio	JAG1	AD	De novo	chr20:10,471400–13,459,333; 3MB heterozygous deletion	N/A	N/A	Alagille syndrome (OMIM# 118450)	Avoidance of intraoperative cholangiogram
30	Trio	NF1	AD	De novo	chr17:29653118	c.5118delT	p.Val1707PhefsTer3	Neurofibromatosis type 1 (OMIM# 162200)	Enlistment of additional subspecialists
		MYBPC3	AD	Inherited (maternal)	chr11:47355113	c.3184delG	p.Val1062LeufsTer13	Cardiomyopathy (OMIM# 615396)	Medication Change
82	Quad	KMT2D	AD	Inherited	chr12:49444140	c.3228_3230delGAA	p.Lys1077del	Kabuki syndrome (OMIM# 147920)	Enlistment of additional subspecialists
92	Trio	CHD7	AD	De novo	chr8:61774803	c.7879C>T	p.Arg2627Ter	CHARGE syndrome (OMIM# 214800)	Enlistment of additional subspecialists
96	Solo	FOXF1	AD	De novo	chr16:86544363	c.188G>T	p.Ser63Ile	Alveolar capillary dysplasia with misalignment of pulmonary veins (OMIM# 265380)	Avoidance of lung biopsy. Transfer to pulmonary transplant center
100	Trio	ZEB2	AD	De novo	chr2:145161633	c.656delG	p.Gly219AlafsTer5	Mowat–Wilson syndrome (OMIM# 235730)	Enlistment of additional subspecialists
108	Trio	TSC2	AD	De novo	chr16:2108829	c.935_936delTC	p.Leu312GlnfsTer25	Tuberous sclerosis-2 (OMIM# 613254)	Targeted genetic counseling (TSC2 more severe phenotype than TSC1)

Most mutations were autosomal dominant and de novo. Five of the mutations were inherited: four were AD and one was XLD. Most were point mutations. Only one child had a structural variant in JAG 1 associated with Alagille syndrome. The disease associations ranged from syndromes like Coffin–Siris syndrome that affected multiple organ systems to disorders limited to the cardiovascular system like LV noncompaction syndrome. Effect on management ranged from enlistment of additional subspecialists to the care of the infant, listing for cardiac transplantation, avoidance of intraoperative cholangiogram to palliative care.

^a^Data on six of these probands were also communicated in a previous publication by our group^14^.

Proband 18 presented with arrhythmias, cardiomegaly, and heart failure. Echocardiography revealed a bicuspid aortic valve and atrial septal defect. rWGS identified two likely pathogenic mutations in *POLR1C*, associated with hypomyelinating leukodystrophy, type 11^14^. This diagnosis could not initially be confirmed since she had received a pacemaker that precluded brain magnetic resonance imaging (MRI). Brain computed tomography was expected to be unrevealing in the neonatal period. It was performed at 1 year of age, revealing white matter hypoplasia or hypomyelination. At 16 months of age, she had not yet developed dentition, was unable to sit and was nonverbal, confirming the molecular diagnosis^16^.

Proband 24 had recurrent hypophosphatemia requiring frequent phosphate supplementation. This had been considered iatrogenic given prolonged diuretic therapy and inability to optimize nutrition. Diagnosis of XLD hypophosphatemic rickets, however, provided a treatable etiologic diagnosis that had not been considered^14^.

### Genetic diagnosis and impact on surgical care

In five probands (12, 20, 26, 30, 96), diagnosis of genetic diseases by rWGS informed surgical care as well as medical management. Proband 12 was diagnosed with Coffin–Siris syndrome after a protracted medical course. At the time of consent, the acuity of her illness worsened from stable ventilation via tracheostomy and tolerance of gastric tube feedings to ventilation via oscillator, fasting, inotropic support, broad-spectrum antibiotics for suspected endocarditis, and consideration for extracorporeal membrane oxygenation (ECMO). Upon diagnosis of Coffin–Siris syndrome the family elected comfort care, and the patient was compassionately extubated^14,17^. Molecular diagnosis of left ventricular noncompaction (LVNC) syndrome implied that proband 20 had a condition limited to the cardiovascular system, which led to more confident early listing for cardiac transplantation^14^. Echocardiographic and MRI findings were inconclusive for LVNC disease. The decision to list for cardiac transplantation was reinforced by published data of better long-term outcome in cardiac transplantation when performed in the neonatal/infant period, when necessary^18^. In proband 26, an intraoperative cholangiogram and possible Kasai hepatoportoenterostomy were canceled in the operating room upon communication of a diagnosis of Alagille syndrome^14^.

Neurofibromatosis and *MYBPC3-*associated cardiomyopathy in proband 30 were diagnosed by rWGS after 8 months of hospitalization complicated by ECMO, multiple infectious workups, chronic respiratory failure leading to tracheostomy, feeding intolerance requiring gastrostomy tube feeds, hypertension, and persistent heart failure. Diagnosis of neurofibromatosis resulted in enlistment of additional subspecialists, including nephrology, for further evaluation and management of the patient’s persistent hypertension. This led to discontinuation of an angiotensin converting enzyme inhibitor drug, which was relatively contraindicated by the risk of neurofibromatosis-associated renal vascular stenosis. The diagnosis also guided the timing of subsequent interventions. Given the increased risk of anesthesia and the potential neurodevelopmental abnormalities associated with neurofibromatosis, subsequent cardiac surgical interventions were delayed until after infancy.

Molecular diagnosis of alveolar capillary dysplasia with misalignment of pulmonary veins in proband 96 precluded the need for a lung biopsy, avoiding the risks of neonatal anesthesia and surgery^19,20^. If rWGS had been performed earlier in the hospitalization of proband 92, a diagnosis of CHARGE syndrome would have changed surgical management. He received supraglottoplasty 1 day prior to rWGS results and tracheostomy 3 weeks later. Earlier diagnosis would have likely lead to earlier referral for tracheostomy given multiple failed extubations, paradoxical vocal cord movement, substantial salivary pooling, and a significant rate of supraglottoplasty failure in patients with CHARGE syndrome with the preceding characteristics^21–24^.

### Genetic diagnosis and familial implications

For three families, the rWGS diagnoses had implications for additional family members. Hypophosphatemic rickets in proband 24 was maternally inherited. This prompted endocrine evaluation of the patient’s mother, which revealed that the patient’s maternal uncle may also be affected, given a history of steroid treatment for unexplained short stature in adolescence. Proband 30 was diagnosed with neurofibromatosis type 1 (*NF1*)^14^, which likely explained his structural heart defect, pulmonary atresia with intact ventricular septum given that the most common cardiac anomaly seen in *NF1* is pulmonary valve anomalies^25^. Proband 30 had a second diagnosis—a frameshift variant in *MYBPC3* associated with cardiomyopathies—that likely explained the proband’s significant heart failure requiring persistent afterload reduction. The *MYBPC3* variant was maternally inherited, prompting referral to cardiac screening of the patient’s asymptomatic mother given the variable expressivity of *MYBPC3-*associated cardiomyopathy^26–29^. Proband 82 was diagnosed with Kabuki syndrome. Family testing also diagnosed a sibling and the father, both with structurally dissimilar CHD, with the same syndrome.

### Genetic diagnosis and implication for additional organ systems

rWGS diagnosis had implications for neurodevelopment in 9 of the 11 (82%) diagnosed probands and had endocrine, immunologic and/or infectious disease implications likely to impact short- and long-term outcomes in 6 of the 11 (52%) (Supplementary Table 6).

### rWGS and healthcare costs

The cost of rWGS, ~$8500 (Supplementary Table 1), has limited its routine use in standard medical care. In this cohort, however, the average total cost of hospitalization was greater than $900,000. This included multiple negative or minimally informative studies and multiple subspecialist consultations in attempts to diagnose and treat the patients. The average daily hospital cost (*p* = 0.19, mean difference −903.4, 95% CI −2176.0, 369.1), average daily physician cost (*p* = 0.12, mean difference −400.5, 95% CI −893.2, 92.3), or average daily total cost of hospitalization (*p* = 0.14, mean difference −1303.9, 95% CI −2999.6, 391.8) did not differ between diagnostic and nondiagnostic rWGS, suggesting that rWGS-based evaluation was cost neutral (Supplementary Table 2a).

### Health care expenditures around rWGS implementation

Implementation of a new intervention like rWGS to medical care requires careful scrutiny. It is possible that the effect of the intervention, here rWGS, may not be reflected in reduced health care expenditures immediately, but rather increased utilization of certain services resulting in higher health care expenditures. Analysis of trends in health care expenditures can assist in the investigation of the effects of an intervention on health care expenditures.

In order to explore the healthcare expenditure trend around the implementation of rWGS, we compared the patterns of spending prior to blood collection for rWGS (period 1), while awaiting rWGS results (period 2) and after rWGS results were known (period 3), i.e., date of admission (DOA) to date of blood collection (DOBC) for rWGS (period 1), DOBC to date of rWGS result (DOR) (period 2), and DOR to date of discharge (DODC) (period 3). Twenty patients had complete spending data for these time periods. Four patients were either discharged or died prior to rWGS results.

**Fig. 3:**
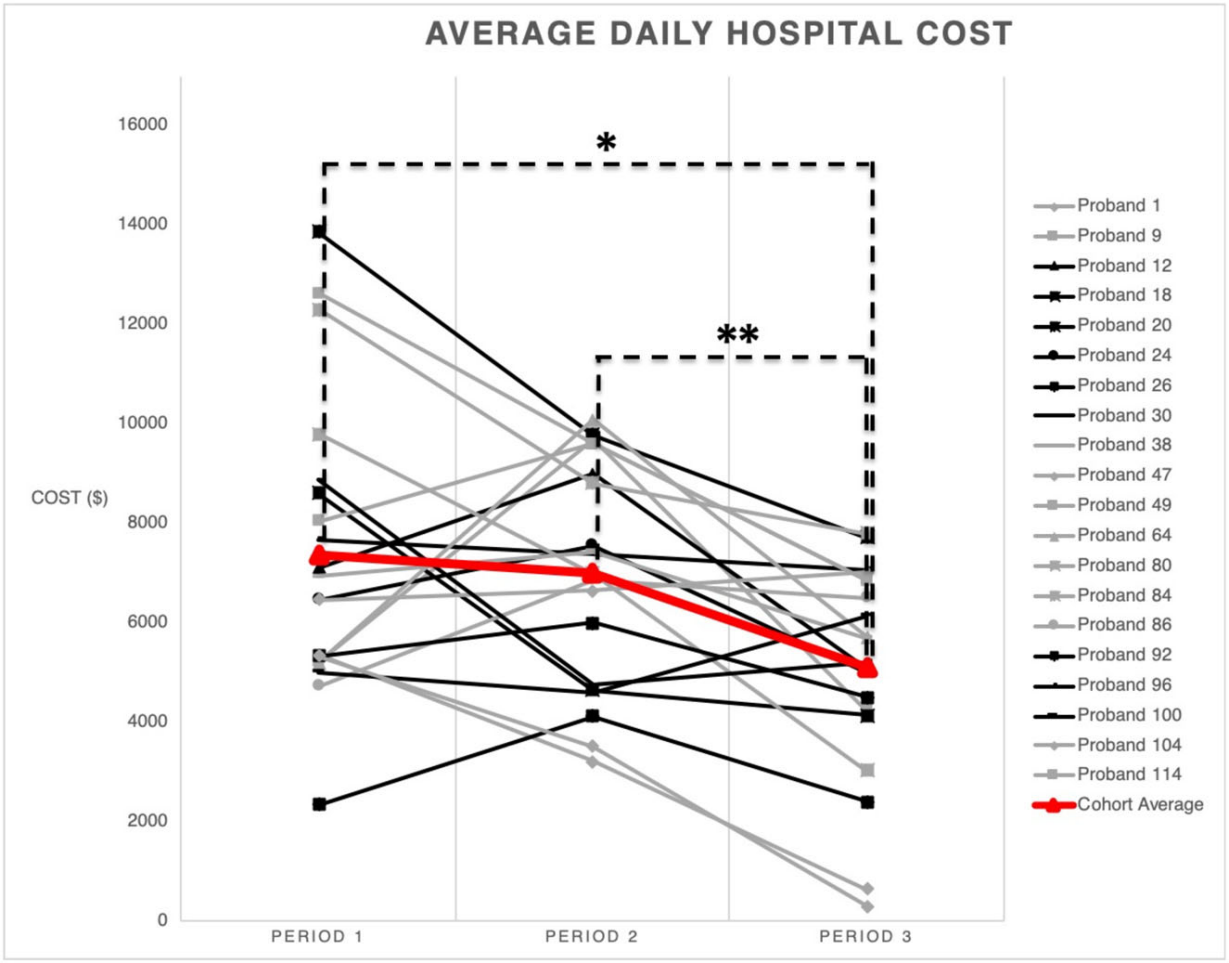
Temporal trends in hospital costs around the time of rWGS testing. Evaluation of spending trend surrounding the rWGS process showed an overall decreased spending post rWGS results (Supplementary Table 2b). There is a significant association between time period and cost (*p* = 0.01; repeated measures ANOVA). Specifically, there is a significant difference in cost between periods 1 and 3 (mean difference 2266.4; 95% CI 1035.6–3497.1; *p = 0.001; paired *t*-test) and periods 2 and 3 (mean difference 1917.1; 95% CI 1077.2–2756.9; **p = 0.0001; paired *t*-test). There is not a significant difference in cost between the nondiagnostic and diagnostic groups by time period (*p* = 0.70; repeated measures ANOVA).

For the 20 patients with complete spending data during these time periods, a significant difference was detected in average daily hospital cost between periods 1 and 3 (*p* = 0.003, mean difference 2266.4, SE 588, 95% CI 722.7, 3810) and periods 2 and 3 (*p* = 0.000, mean difference 1917.1, SE 401.3, 95% CI 863.7, 2970.4), showing decreased spending after rWGS results were known (Fig. 3, Supplementary Table 2b).

There was no significant difference in spending during the time period prior to blood collection for rWGS (period 1) and the time period while awaiting rWGS results (period 2), periods 1 and 2, (*p* = 1, mean difference 349.29, SE 614.78, 95% CI −1264.57, 1963.15) (Fig. 3, Supplementary Table 2b). Spending in period 2 was dichotomous: 50% (*n* = 10) of the cohort had increased cost relative to period 1, while 50% (*n* = 10) had decreased cost. One explanation for this finding is the higher frequency of major surgical procedures, cardiac and other, in patients with increased cost during this period (60%) compared with the group with decreased cost (30%); however, this difference did not reach statistical significance likely due to sample size. There was no difference in the number of patients who underwent expected cardiac surgical procedures in the two groups (Supplementary Table 7). Costs during this time were also driven by noncardiac procedures and reintubations/mechanical ventilation.

The overall spending in the period after knowledge of rWGS results, period 3, was down trending for the whole cohort (Fig. 3, Supplementary Table 2b). There was no significant difference in hospital cost between the nondiagnostic and diagnostic groups by time period (*p* = 0.70; repeated measures ANOVA) (Supplementary Table 2a).

### Healthcare expenditures by tercile costs of hospitalization

**Fig. 4:**
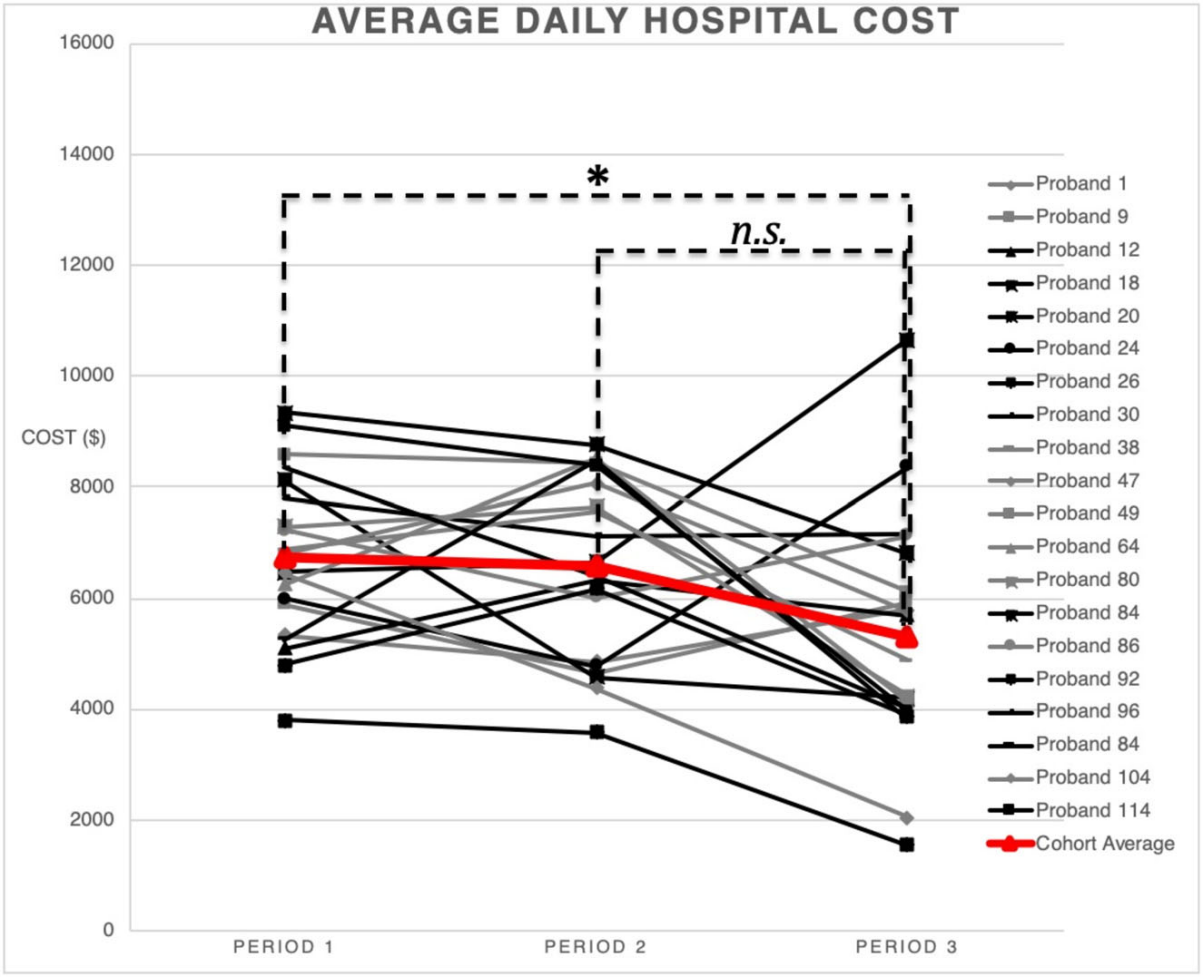
Average daily hospital cost per tercile of hospitalization. Total Hospital cost was divided in three equal parts and the average daily hospital costs calculated (Supplementary Table 2c). There is a significant association between time period and cost (*p* = 0.047; repeated measures ANOVA). There was statistically significant decrease in average daily hospital cost from the first third of the hospitalization compared to the last third (*p* = 0.036, mean difference 1438.53, SE 518.81, 95% CI 76.60–2800.47), but there was no statistically significant decrease when comparing the first third of the hospitalization with the second third (*p* = 1, mean difference 178.22, SE 352.18, 95% CI −746.30 to 1102.73) or the second third with the last third (*p* = 0.1, mean difference 1260.32, SE 551.59, 95% CI −187.66 to 2708.30*;* repeated measures ANOVA with Bonferroni correction).

To account for a natural trend in decreasing hospital cost over time as patients neared the end of hospitalization an analysis was done comparing the tercile costs of the hospitalization. There was a significant decrease in cost between the first third of the hospitalization (tercile 1) and the last third (tercile 3) (*p* = 0.036, mean difference 1438.5, SE 518.8, 95% CI 76.6, 2800.5) as expected, but this decrease was less than the decrease in spending seen around rWGS implementation between periods 1 and 3 and periods 2 and 3. There was no statistically significant difference between terciles 1 and 2 (*p* = 1) or terciles 2 and 3 (*p* = 0.1) (Fig. 4, Supplementary Table 2c).

## Discussion

Rapid WGS has been described as a powerful diagnostic tool in multiple intensive care settings^10,14,30,31^. The field continues to investigate different areas to help guide implementation of rapid WGS including the benefits of singleton versus trio testing^10,30^, best platforms^32^, and, ultimately, the clinical and economic utility of this testing^10,14,31^. Furthermore, determining which patient population would benefit from this comprehensive genome testing approach is critical to ensure resources are optimally utilized.

CHD is the most common and a costly congenital disorder and can be associated with significant long-term functional impairment. In addition to the cardiovascular defect some patients also tend to have other organ system involvement. While additional structural congenital anomalies are relatively easier to ascertain, immune and endocrine system effects may not be evaluated until late in the course of illness. Furthermore, neurodevelopmental disorders are infrequently ascertained in early infancy. Particular effort is made to understand and improve neurodevelopmental outcomes in children with CHD. It is imperative to understand the underlying etiology of disease in order to provide optimal care.

Children with CHD and genetic syndromes are at increased risk of surgical morbidity and mortality compared to other children with isolated CHD^9^. Knowledge of the child’s underlying genetic condition not only helps the clinician minimize risks but also allows for more informed discussions with parents prior to medical and surgical interventions. This was evident in the care of an infant who was ultimately diagnosed with Coffin–Siris syndrome after prolonged hospitalization with multiple complications including ECMO, frequent infections, and multiple surgical interventions^17^.

A frequent difficulty in making a genetic diagnosis and limitation to phenotype driven gene/gene panel testing in the newborn is the incomplete phenotype relative to textbook descriptions of disease. This was evidenced by the fact that the incorrect genetic test was recommended for three of the seven patients (43%) who received formal clinical genetics consultations. For example, the newborn diagnosed with X-linked hypophosphatemic rickets syndrome would likely not have been diagnosed until the appearance of leg bowing at weight-bearing age. Rickets diagnosis after 5 years of age is associated with increased fractures, increased surgeries, and final growth percentile of <10^th^ (most <3^rd^)^33–35^. This diagnosis also informed familial decision-making given X-linked inheritance.

The presentation of Kabuki syndrome is very subtle in the neonatal period and can be easily missed. However, this syndrome is associated with much morbidity. Knowing that these patients can have impaired immune systems prior to surgical intervention facilitates preoperative management and can improve outcomes. A diagnosis of Kabuki syndrome may demand preoperative treatment with intravenous immunoglobulin in those with clinically significant hypogammaglobulinemia and can inform appropriate perioperative antibiotic prophylaxis^36,37^. Moreover, it is not unusual for parents to be diagnosed with a milder form of a genetic disease when a more affected child receives a diagnosis. The father diagnosed with Kabuki syndrome herein had a constellation of anomalies that in retrospect could have led to a clinical diagnosis. Identification of the same disorder in his children, who had more severe cardiac phenotypes but milder facial phenotype, is a poignant example of the benefits of genomic sequencing in the neonatal period.

Benefits of early diagnosis were also observed in a neonate with *NF1*, where rWGS results led to early involvement of neurologic and renal specialty care and discontinuation of a potentially nephrotoxic antihypertensive agent given increased risk of renal artery stenosis. The café-au-lait spots pathognomonic of this disease were not apparent in this patient until 7 weeks after rWGS diagnosis.

Knowledge of underlying genetic diseases in CHD can lead to more effective healthcare provision. For example, patients with Williams syndrome (chromosome 7q11 deletion) have a high risk for anesthesia-related adverse events. This has led to anesthesia protocols geared specifically to minimize the risk for these patients^38–40^. Several of the cases reported in this study demonstrated that genomic testing should occur early in the hospitalization of infants with structural CHD if it is to optimally influence management decisions. rWGS led to more diagnoses in our cohort compared to CMA and gene panel testing. It is possible that a comprehensive CHD gene panel would have led to a diagnosis in some of the cases, but potential diagnosis would have been limited to the genes incorporated on the gene panel. Underlying genetic conditions currently not associated with CHD would be missed. For example, proband 30 may have been able to obtain a diagnosis of *MYBPC3*-associated cardiomyopathy through a comprehensive CHD gene panel but the diagnosis of *NF1* would not have been made.

The high diagnosis rate by rWGS in this study could be due to patient selection/referral by the inpatient medical team. All our patients were inpatients, critically ill, and most had either additional anomalies or a medical course that deviated from the norm leading to prolonged hospitalization in some. We saw an overall decrease in average daily hospital costs with the implementation of rWGS irrespective of whether a diagnosis was made. The cost neutrality could be a reflection of the fact that most patients were enrolled late in their hospitalization, when rWGS became available or due to potential covariates/confounders not investigated in this study. More data are necessary to determine barriers to implementation of genomic sequencing and its cost-effectiveness in the care of the infant with structural CHD given the small sample size of our study.

Genomic testing should be pursued early in the management of critically ill infants with structural CHD given the myriad of potential genetic diagnoses. rWGS lead to more diagnoses than CMA and gene panel testing in critically ill children with structural CHD. Furthermore, rWGS provided timely actionable information that impacted the care received by these infants. The cost of rWGS was not prohibitive to its implementation in the care of this cohort of infants and there is a strong signal that rWGS leads to decreased hospital spending in this patient population.

## Methods

### Study design

Retrospective comparison of clinical utility, outcomes, and healthcare utilization of rWGS and clinical genetic testing was approved by the institutional review board (IRB) at Rady Children’s Hospital-San Diego (RCHSD)/University of California-San Diego (ClinicalTrials.gov NCT02917460) and the Food and Drug Administration. Inpatient infants at RCHSD without etiologic diagnoses, and in whom a genetic disorder was possible, were nominated by diverse clinicians from July 25, 2016 to June 28, 2017. Informed written consent was obtained from at least one biological parent or guardian. The acute clinical utility of rWGS-based diagnoses (i.e., short-term implementation of precision medicine interventions) and impact on outcomes were evaluated by electronic medical records (EMR) review, interviews with clinicians, published values, and evaluation by at least two pediatricians, of whom one was a relevant pediatric subspecialist and one a medical geneticist. The length of hospital stay, actual physician worked relative value units, and cost of inpatient care were measured. Facility costs are estimated by multiplying hospital charges by the estimated cost to charge ratio supplied by Rady Children’s Hospital Chief Financial Officer. Professional costs are estimated by multiplying professional charges by the estimated average payment to charge ratio for professional services^14^ (Supplementary Table 1).

### rWGS, interpretation, and reporting

The analysis method for rapid genomic sequencing has been previously described^10,14,30^. Blood samples from inpatient infants were obtained within the maximum allowable daily phlebotomy and minimum hemoglobin in infants with respiratory or cardiovascular compromise. Blood samples were obtained from probands and parents (trios), where possible. Deoxyribonucleic acid was isolated using standard methods and WGS libraries were prepared with polymerase chain reaction-free methods (Illumina, San Diego, CA) as described^41^. rWGS was performed at Envision Inc. (Huntsville, AL) for the first nine families by 47-fold 2 × 150 nucleotide (nt) sequencing on Illumina HiSeq X instruments (5–10 days turnaround). Remaining families were sequenced in house in two modes: in very ill infants, 2 × 100 nt proband rWGS was performed on HiSeq 2500s in rapid run mode. Other rWGS was 2 × 150 nt on a HiSeq 4000^42^. Rapid alignment and variant calling was done by Dragen (Edico Genome, San Diego, CA; Supplementary Table 3)^41^. Variants were annotated, analyzed, and interpreted with Opal Clinical (Fabric Genomics, Oakland, CA)^43,44^. Clinical features of infants were manually extracted from EMR, translated into human phenotype ontology terms (Supplementary Table 4), mapped to all known genetic diagnoses and associated disease causing genes, and a phenotypic-specific gene list was generated by Phenolyzer^45,46^. In addition, variants were also prioritized by phenotype using VAAST and Phevor via the Fabric Genomics interpretation platform^47^. Briefly, multiple filtering protocols were used to analyze each rapid WGS case. First, two phenotypic-driven protocols were used (1) ranking the variants using the VAAST and Phevor algorithm and (2) filtering variants using a patient-specific gene list generated by Phenolyzer. In addition, multiple phenotype-agnostic filters were applied including various inheritance models (de novo, dominant, recessive, X-linked), loss-of-function variants, and variants found in specific database such as ClinVar and HGMD. Variants were selected for curation and classified based on ACMG guidelines^32^. Only likely pathogenic and pathogenic variants were reported based on IRB guidance. All reports were approved by board-certified molecular geneticists. Causative variants were confirmed by Sanger sequencing.

### Healthcare expenditure trends evaluation

Healthcare expenditure trend around the implementation of rWGS was evaluated by comparing the patterns of spending prior to blood collection for rWGS (period 1), while awaiting rWGS results (period 2) and after rWGS results were known (period 3). Equal tercile costs of the hospitalization were also analyzed as a control for the natural trend of decreased spending seen toward the end of a hospitalization. Hospital and physician cost data for all time periods were provided by the business office at Rady Children’s Hospital for statistical analysis. The date range for each time period of interest, DOA to DOBC for rWGS (period 1), DOR (period 2), and DODC (period 3), was identified for each patient. The total physician and hospital cost for that particular period was calculated and averaged over the total number of days spanning that period for each patient to determine the average daily spending over that time period. Statistical analyses were performed by a statistician as described in the manuscript to comparing spending between the time periods. Tercile cost was calculated by dividing the total hospital stay in three equal time periods. The total physician and hospital cost for that particular time period were calculated and averaged over the number of days spanning that period to determine the average daily spending over that time period. Statistical analyses were performed as described in the manuscript to compare the spending between the time periods (Supplementary Table 2a–c).

### Statistical analysis

Fisher’s exact test was used for analysis of categorical variables. Wilcoxon rank sum test was used for nonnormally distributed continuous variables. McNemar’s test with continuity correction was used to assess differences in diagnostic rates between rWGS, CMA, and/or targeted gene panels. The confidence intervals were calculated according to Wilson and Sheskin^48,49^. Overall, trends in average daily hospital cost were evaluated with repeated measures ANOVA and Bonferroni correction. Analyses were performed in either R v3.5.211 or SPSS v. 2612^50,51^. Two-tailed *p* values less than 0.05 were considered statistically significant.

### Reporting summary

Further information on research design is available in the Nature Research Reporting Summary linked to this article.

## Supplementary information

Reporting Summary

Supplementary Information

## Data Availability

All data associated with this study are present in the paper or are available at the Longitudinal Pediatric Data Resource under a data use agreement and subject to the limitations of the informed consent documents for each subject (accession number nbs000003.v1.p, https://nbstrn.org/tools/lpdr).
