# Author Correction: Rapid whole genome sequencing impacts care and resource utilization in infants with congenital heart disease

**DOI:** 10.1038/s41525-021-00205-9

**Published:** 2021-05-26

**Authors:** Nathaly M. Sweeney, Shareef A. Nahas, Shimul Chowdhury, Sergey Batalov, Michelle Clark, Sara Caylor, Julie Cakici, John J. Nigro, Yan Ding, Narayanan Veeraraghavan, Charlotte Hobbs, David Dimmock, Stephen F. Kingsmore

**Affiliations:** 1 Rady Children’s Institute for Genomic Medicine, San Diego, CA USA; 2 Rady Children’s Hospital, San Diego, CA USA; 3 Department of Pediatrics, University of California San Diego, La Jolla, CA USA; 4 Department of Family Medicine and Public Health, University of California San Diego, La Jolla, CA USA; 5 Department of Surgery, University of California San Diego, La Jolla, CA USA

**Keywords:** Health care economics, Medical genomics, Congenital heart defects

Correction to: *npj Genomic Medicine* 10.1038/s41525-021-00192-x, published online 22 April 2021

The original version of this Article contained errors in the Reference list order. The order of References has been corrected in the HTML and PDF versions of the Article.

