# Supplementary material for: Rapid whole genome sequencing impacts care and resource utilization in infants with congenital heart disease
Source: NPJ Genom Med. 2021 Apr 22;6:29. doi: 10.1038/s41525-021-00192-x (PMC8062477; doi:10.1038/s41525-021-00192-x)
Supplement: Supplementary file 1 — Reporting Summary [file 41525_2021_192_MOESM1_ESM.pdf]

nature research

Corresponding author(s): Nathaly M. Sweeney

Last updated by author(s): Nov 18, 2020

Reporting Summary

Nature Research wishes to improve the reproducibility of the work that we publish. This form provides structure for consistency and transparency in reporting. For further information on Nature Research policies, see our Editorial Policies and the Editorial Policy Checklist.

Statistics

For all statistical analyses, confirm that the following items are present in the figure legend, table legend, main text, or Methods section.

n/a	Confirmed	
☐	☒	The exact sample size (*n*) for each experimental group/condition, given as a discrete number and unit of measurement
☒	☐	A statement on whether measurements were taken from distinct samples or whether the same sample was measured repeatedly
☐	☒	The statistical test(s) used AND whether they are one- or two-sided<br>*Only common tests should be described solely by name; describe more complex techniques in the Methods section.*
☒	☐	A description of all covariates tested
☐	☒	A description of any assumptions or corrections, such as tests of normality and adjustment for multiple comparisons
☐	☒	A full description of the statistical parameters including central tendency (e.g. means) or other basic estimates (e.g. regression coefficient) AND variation (e.g. standard deviation) or associated estimates of uncertainty (e.g. confidence intervals)
☐	☒	For null hypothesis testing, the test statistic (e.g. *F*, *t*, *r*) with confidence intervals, effect sizes, degrees of freedom and *P* value noted<br>*Give P values as exact values whenever suitable.*
☒	☐	For Bayesian analysis, information on the choice of priors and Markov chain Monte Carlo settings
☒	☐	For hierarchical and complex designs, identification of the appropriate level for tests and full reporting of outcomes
☒	☐	Estimates of effect sizes (e.g. Cohen's *d*, Pearson's *r*), indicating how they were calculated

Our web collection on statistics for biologists contains articles on many of the points above.

Software and code

Policy information about availability of computer code

Data collection	No software was used
Data analysis	Dragen (Edico Genome, San Diego, CA), Opal Clinical (Fabric Genomics, Oakland, CA), Phenolyzer, VAAST and Phevor via the Fabric Genomics interpretation platform

For manuscripts utilizing custom algorithms or software that are central to the research but not yet described in published literature, software must be made available to editors and reviewers. We strongly encourage code deposition in a community repository (e.g. GitHub). See the Nature Research guidelines for submitting code & software for further information.

Data

Policy information about availability of data

All manuscripts must include a data availability statement. This statement should provide the following information, where applicable:

- Accession codes, unique identifiers, or web links for publicly available datasets
- A list of figures that have associated raw data
- A description of any restrictions on data availability

Data are available at LPDR (https://www.nbstrn.org/research-tools/ longitudinal-pediatric-data-resource).

Field-specific reporting

Please select the one below that is the best fit for your research. If you are not sure, read the appropriate sections before making your selection.

☒ Life sciences ☐ Behavioural & social sciences ☐ Ecological, evolutionary & environmental sciences

For a reference copy of the document with all sections, see nature.com/documents/nr-reporting-summary-flat.pdf

Life sciences study design

All studies must disclose on these points even when the disclosure is negative.

Sample size	N/A
Data exclusions	N/A
Replication	N/A
Randomization	N/A
Blinding	N/A

Reporting for specific materials, systems and methods

We require information from authors about some types of materials, experimental systems and methods used in many studies. Here, indicate whether each material, system or method listed is relevant to your study. If you are not sure if a list item applies to your research, read the appropriate section before selecting a response.

Materials & experimental systems

n/a	Involved in the study	
☒	☐	Antibodies
☒	☐	Eukaryotic cell lines
☒	☐	Palaeontology and archaeology
☒	☐	Animals and other organisms
☐	☒	Human research participants
☐	☒	Clinical data
☒	☐	Dual use research of concern

Methods

n/a	Involved in the study	
☒	☐	ChIP-seq
☒	☐	Flow cytometry
☒	☐	MRI-based neuroimaging

Human research participants

Policy information about studies involving human research participants

Population characteristics	Inpatient infants (age 0-11 months) at RCHSD without etiologic diagnoses, and in whom a genetic disorder was possible. Sixty-seven percent of the patients who underwent rWGS were male, 34% were females. Families predominantly identified enrolled infants as Hispanic/Latino (42%), 25% Caucasian, 12% African/African American, 8% Asian/Native American/Pacific Islander, 12% other.
Recruitment	Inpatient infants at RCHSD without etiologic diagnoses, and in whom a genetic disorder was possible, were nominated by diverse clinicians from July 25, 2016–June 28, 2017. Informed consent was obtained from at least one biological parent, or guardian.
Ethics oversight	This study was approved by the institutional review board (IRB) at Rady Children’s Hospital- San Diego (RCHSD) and the Food and Drug Administration (FDA).

Note that full information on the approval of the study protocol must also be provided in the manuscript.

Clinical data

Policy information about clinical studies

All manuscripts should comply with the ICMJE guidelines for publication of clinical research and a completed CONSORT checklist must be included with all submissions.

Clinical trial registration	ClinicalTrials.gov NCT02917460

Study protocol	ClinicalTrials.gov NCT02917460
Data collection	Rady Children's Hospital San Diego from July 25, 2016–June 28, 2017
Outcomes	Rate of genetic diagnosis using rWGS compared to rate of diagnosis using microarray and or gene panels. Healthcare expenditure around the implementation of rWGS. We evaluated the trend in healthcare expenditure based on rWGS status surrounding three time periods: 1. prior to sample collection, 2. after sample collection-before results, 3. after results.
