# Supplementary material for: Rapid whole genome sequencing impacts care and resource utilization in infants with congenital heart disease
Source: NPJ Genom Med. 2021 Apr 22;6:29. doi: 10.1038/s41525-021-00192-x (PMC8062477; doi:10.1038/s41525-021-00192-x)
Supplement: Supplementary file 2 — Supplementary Information [file 41525_2021_192_MOESM2_ESM.pdf]

Supplementary Materials

Rapid Whole Genome Sequencing Impacts Care and Resource Utilization in Infants with Congenital Heart Disease

Authors: Nathaly M. Sweeney MD, MPH[1,2,3], Shareef A. Nahas PhD[1], Shimul Chowdhury PhD[1], Sergey Batalov MS[1], Michelle Clark PhD[1], Sara Caylor BSN[1], Julie Cakici BSN[1], John J. Nigro MD[2,4], Yan Ding MD[1], Narayanan Veeraraghavan PhD[1], Charlotte Hobbs, MD PhD[1], David Dimmock MD[1], Stephen Kingsmore MB ChB BAO DSc[1]

Affiliations:

1. Rady Children's Institute for Genomic Medicine, San Diego, CA, USA;
2. Rady Children's Hospital, San Diego, CA, USA
3. Department of Pediatrics, University of California San Diego, San Diego, CA, USA;
4. Department of Surgery, University of California San Diego, San Diego, CA, USA

Correspondence: N. Sweeney (nmsweeney@health.ucsd.edu), (858)966-5818. Rady Children's Institute for Genomic Medicine, Rady Children's Hospital, 3020 Children's Way, San Diego, CA 92123, USA

The RCIGM investigators are Matthew Bainbridge, Jeanne Carroll, Casey Cohenmeyer, Marva Evans, Lauge Farnaes, Jennifer Friedman, Joseph Gleeson, Amber Hildreth, Jose Honold, Amy Kimball, Brian Lane, Crystal Le, Sandra Leibel, Laurel Moyer, Lawrence Prince, Mark Speziale, Charles Sauer, Denise Suttner, Richard Song, Lisa Salz, Kristen Wigby, Audra Wise, Meredith Wright.

Supplementary Materials

Supplementary Data Page 2
Supplementary Table 1: Unit Cost Analysis of rWGS Page 2
Supplementary Table 2a: Cost of hospitalization in the rWGS group Page 3
Supplementary Table 2b: Cost of hospitalization in the rWGS group Page 4
Supplementary Table 2c: Cost of hospitalization in the rWGS group Page 5
Supplementary Table 3: Human Phenotype Ontology terms for clinical features in probands Page 6
Supplementary Table 4: Metrics of rapid whole genome sequencing for probands Page 7
Supplementary Table 5: Cardiac defects, rWGS and clinical genetic tests Page 12
Supplementary Table 6: Neurodevelopmental/Infectious/Immunological and Endocrinological implications of rWGS diagnosis Page 15
Supplementary Table 7; Cardiac defects, cardiac surgical interventions, Cost trends from DOBC to DOR Page 17
Supplementary References Page 25

Supplementary Data

Supplementary Table 1: Unit Cost Analysis of rWGS.

	Singleton	Trio	Duo	Quad
**Direct Costs**				
Labor				
Enrolment/Intake				
Fellow	$31	$31	$31	$31
MD	$154	$154	$154	$154
Genomics Employees	$2,186	$3,279	$2,733	$3,826
Total Labour	$2,371	$3,464	$2,918	$4,011
Non-Labor				
IT	$604	$1,813	$1,208	$2,417
Sequencing	$2,898	$8,694	$5,796	$11,592
Data Analysis	$386	$1,159	$773	$1,546
Reporting	$150	$150	$150	$150
Total Non-Labour	$4,039	$11,816	$7,927	$15,704
**Indirect Costs**				
Genomics Non-Lab Employees & Professional fees	$892	$892	$892	$892
Supplies	$42	$42	$42	$42
Purchased services	$298	$298	$298	$298
Leases and rentals	$287	$287	$287	$287
Depreciation	$454	$680	$567	$794
Other expenses	$100	$100	$100	$100
Total Indirect Costs	$2,072	$2,299	$2,186	$2,412
**Total Cost per Infant**	$8,482	$17,579	$13,031	$22,128
(5 singletons, 16 trios, 2 duos, 1 quad)	$42,410	$281,264	$26,062	$22,128
**Total Cost for 24 enrolled families**	**$371,864**			

The full cost of rWGS as a CLIA Laboratory Developed Test, including indirect costs and treatment guidance, was $8482–$22,128 (singleton, duo, trio, and quad). The total cost of rWGS in 24 families was $371,864. Included were consultation for pretest assessment, counseling, result disclosure, precision medicine guidance, and trio Sanger sequencing to validate variants detected by rWGS.

Supplementary Table 2a: Cost of hospitalization in the rWGS group

Patient	Cost ($)					
	Hospital	Physician	Total	Daily Hospital (Avg)	Daily Physician (Avg)	Daily total (Avg)
1	304,392	116,521	42,0913	3,853.06	1,474.95	5,328.01
9	863,890	331,984	1,195,874	3,962.80	1,522.86	5,485.66
*12	1,424,903	421,070	1,845,973	5,699.61	1,684.28	7,383.89
*18	505,784	123,235	629,019	4,515.93	1,100.31	5,616.24
*20	1,712,741	401,737	2,114,478	6,414.76	1,504.63	7,919.39
*24	813,837	296,545	1,110,382	4,677.22	1,704.28	6,381.51
*26	27,973	8,070	36,043	2,543.00	733.64	3,276.64
*30	2,380,629	667,178	3,047,807	5,750.31	1,611.54	7,361.85
38	1,415,016	446,997	1,862,013	4,896.25	1,546.70	6,442.95
47	1,070,163	374,235	1,444,398	5,096.01	1,782.07	6,878.09
49	2,077,057	684,939	2,761,996	5,785.67	1,907.91	7,693.58
64	275,511	129,936	405,447	4,238.63	1,999.02	6,237.65
65	135,011	50,973	185,984	5,870.04	2,216.22	8,086.26
80	464,375	141,044	605,419	6,361.30	1,932.11	8,293.41
*82	93,146	40,256	133,402	4,435.52	1,916.95	6,352.48
84	569,679	152,871	722,550	6,548.03	1,757.14	8,305.17
86	1,104,726	421,232	1,525,958	4,845.29	1,847.51	6,692.80
*92	582,865	178,898	761,763	3,784.84	1,161.68	4,946.51
*96	449,960	146,821	596,781	4,687.08	1,529.39	6,216.47
*100	108,364	49,254	157,618	4,013.48	1,824.22	5,837.70
104	57,734	17,456	75,190	3,396.12	1,026.82	4,422.94
*108	22,233	10,479	32,712	3,705.50	1,746.50	5,452.00
^112	10,699	4,189	14,888	10,699.00	4,189.00	14,888.00
114	494,115	135,659	629,774	5,551.85	1,524.26	7,076.11
SUM	16,964,803	5,351,579	2,2316,382	121,331.33	41,243.98	162,575.32
AVG	706,866.79	222,982	929,849.25	5,055.47	1,718.50	6,773.97

There was no statistically significant difference between average daily hospital cost (mean difference diagnostic vs non-diagnostic rWGS: -903.4 (-2176.0, 369.1);p=0.19), average daily physician cost (mean difference diagnostic vs non-diagnostic rWGS: -400.5 (-893.2, 92.3);p=0.12) or average daily total cost of hospitalization (mean difference diagnostic vs non-diagnostic: -1303.9 (-2999.6, 391.8);p=0.14) between diagnostic and non-diagnostic rWGS. ^Patient died on hospital day 1. *Diagnostic rWGS. P-values calculated using Wilcoxon rank sum test due to deviations from normality. Avg=Average

Supplementary Table 2b: Average daily total cost of hospitalization in the rWGS group by time period

Patient	Cost ($)		
	Period 1 (DOA-DOBC)	Period 2 (DOBC-DOR)	Period 3 (DOR-DODC)
1	5,347.29	3,206.00	639.00
9	5,221.47	9,693.96	4,242.34
*12	7,103.56	8,982.15	5,067.00
*18	8,602.00	4,649.96	4,132.00
*20	13,862.00	9,777.00	7,721.00
*24	6,466.49	7,559.14	5,017.24
*26	2,344.00	4,115.00	2,402.00
*30	7,673.37	7,379.23	7,075.50
38	6,930.00	7,437.52	5,689.00
47	6,459.00	6,655.00	7,009.00
49	8,044.05	9,586.00	6,863.97
64	5,198.00	10,084.00	5,721.00
80	9,789.00	6,983.00	3,032.00
84	12,294.00	8,813.00	7,795.00
86	4,726.00	6,867.00	6,512.00
*92	5,336.00	6,003.20	4,512.00
*96	8,875.00	4,756.30	5,217.12
100	5,017.00	4,611.00	6,134.00
104	5,343.00	3,522.00	294.00
114	12,626.00	9,591.00	6,855.00
AVG	7,362.86	7,013.57	5,096.51

Spending trend surrounding the rWGS process for the patients with complete data during the time periods of healthcare trend evaluation. There is a significant association between time period and cost (p=.01; repeated measures ANOVA). Specifically, there is a significant difference in cost between periods 1 and 3 (Mean Difference 2266.4; 95% CI 1035.6 - 3497.1;p=0.001;paired t-test) and periods 2 and 3 (Mean Difference 1917.1; 95% CI 1077.2 - 2756.9;p=0.0001;paired t-test). There is not a significant difference in cost between the nondiagnostic and diagnostic groups by time period (p=0.70; repeated measures ANOVA). *Diagnostic rWGS.

Supplementary Table 2c: Average daily total cost of hospitalization in the rWGS group with complete cost data by equal terciles

Patient	Cost ($)		
	Tercile 1	Tercile 2	Tercile 3
1	5,327.65	4,846.65	5,791.48
9	5,897.62	4,633.95	5,931.53
*12	5,099.07	6,305.09	5,694.73
*18	8,118.68	4,576.77	4,191.77
*20	6,463.37	6,645.42	10,649.39
*24	6,001.93	4,786.45	8,356.14
*26	3,796.75	3,584.25	1,537.00
*30	7,799.10	7,120.60	7,166.00
38	6,868.28	7,570.71	4,882.22
47	6,805.93	8,065.13	5,768.77
49	8,581.53	8,418.66	6,107.22
64	6,260.76	8,517.72	4,134.79
80	7,296.79	7,639.58	4,236.08
84	9,344.24	8,762.58	6,808.72
86	7,238.03	6,013.65	7,125.59
*92	4,804.73	6,180.22	3,875.60
96	8,342.68	6,415.35	4,003.29
*100	5,245.78	8,455.67	3,811.67
104	6,450.34	4,370.33	2,053.20
114	9,108.69	8,378.79	3,956.07
AVG	6,742.60	6,564.38	5,304.06

Total Hospital cost divided in three equal parts and the average daily hospital costs calculated. There is a significant association between time period and cost (p=.047; repeated measures ANOVA). There was statistically significant decrease in average daily hospital cost from the first third of the hospitalization compared to the last third (p=0.036, Mean Difference 1438.53, SE 518.81, 95% CI 76.60-2800.47), but there was no statistically significant decrease when comparing the 1st third of the hospitalization with the 2nd third (p=1, Mean Difference 178.22, SE 352.18, 95% CI -746.30-1102.73) or the 2nd third with the last 3rd (p=0.1, Mean Difference 1260.32, SE 551.59, 95% CI -187.66-2708.30; repeated measures ANOVA with Bonferroni correction). *Diagnostic rWGS.

Supplementary Table 3: Metrics of rapid whole genome sequencing for twenty-four proband inpatient infants

Family ID	Raw sequence (GB)	% reads mapped	% duplicates	Yield (GB)	Mean Insert size (nt)	Average coverage	MIM genes with <10X coverage at 100% CD nt	MIM genes with ≥10X coverage at 100% of CD nt	Nt variant calls	Passing nt variant calls	CD nt variants	SNVs	Indels	Nt variant Hom /Het ratio	Ti/Tv ratio (in CD)
1	190.6	99.00%	11.80%	166.5	332.2	50.0	276	98.00%	5060268	4881770	27452	4050165	889492	0.56	2.03
9	163.1	98.70%	10.40%	144.8	341.8	43.0	316	97.80%	4788941	4702331	26445	3934041	854900	0.57	2.04
12	195.5	98.80%	10.00%	174.1	369.3	52.0	254	98.20%	4945287	4867107	27343	4059083	886204	0.53	2.04
18	197.8	98.60%	10.00%	179.5	392.4	49.3	285	98.00%	5083464	4975587	25953	4088583	887004	0.49	1.98
20	144.9	98.40%	4.10%	137.5	407.6	42.0	520	96.30%	4787333	4685966	26064	3913210	874123	0.67	2.03
24	217.8	98.80%	14.90%	183.5	358.3	47.6	315	97.80%	4974795	4862566	26778	3996325	866241	0.55	1.98
26	135.1	98.60%	2.90%	129.9	390.6	40.6	215	98.50%	4763155	4720511	26645	3940960	876245	0.63	2.03
30	275.0	98.90%	25.70%	202.0	351.9	45.8	268	98.10%	4932826	4820090	26800	3956885	863205	0.68	1.96
38	177.6	98.90%	9.10%	175.7	339.4	48.7	337	97.60%	4909747	4794272	26569	3938653	855619	0.57	1.96
47	169.0	98.20%	14.40%	156.0	417.0	43.0	325	97.70%	5111922	5026580	27903	4127940	898640	0.58	1.96
49	155.7	98.80%	7.60%	153.8	395.8	42.1	445	96.80%	4975793	4885594	27524	4030736	854858	0.58	1.94
64	202.7	98.90%	8.20%	200.6	411.5	56.1	239	98.30%	5017220	4876361	27611	4034132	842229	*0.64*	1.91
65	164.5	98.90%	8.60%	162.6	414.7	45.2	348	97.50%	4966944	4829817	27275	4011528	818289	0.56	1.94
80	243.2	73.90%**	13.60%	179.6	417.3	62.4	235	98.34%	5654509	5512543	28372	4561483	951060	0.39	1.93
82	162.8	98.40%	3.00%	160.2	365.3	47.3	394	97.21%	5018475	4911886	24758	4010946	900940	0.56	1.95
84	131.2	98.70%	2.70%	129.5	389.0	38.4	669	95.26%	5559549	5463776	27962	4482940	980836	0.43	1.96
86	167.6	98.80%	9.90%	165.6	450.5	40.9	476	96.63%	5026704	4920452	25952	4077211	843241	0.55	1.93
92	249.2	99.00%	8.50%	246.6	413.8	62.7	230	98.37%	5021492	4893226	24837	4041753	851473	0.57	1.92
96	146.0	98.60%	2.60%	144.1	403.3	41.8	586	95.85%	5853946	5753090	29361	4719925	1033165	0.47	1.96
100	154.5	98.50%	2.70%	152.3	426.7	44.1	533	96.23%	4922578	4821402	24904	3937729	883673	0.59	1.95
104	234.4	99.00%	13.70%	232.0	395.6	52.6	247	98.25%	5594682	5455289	27937	4517661	937628	0.43	1.92
108	151.5	98.80%	8.40%	149.8	418.5	38.1	523	96.30%	4977806	4877602	24928	4060905	816697	0.58	1.92
112	190.8	98.80%	14.60%	188.6	393.5	41.6	379	97.32%	5020358	4904266	25010	4067012	837254	0.59	1.92
114	200.0	98.90%	10.80%	197.8	419.9	48.0	245	98.26%	4978561	4838703	24457	3999455	839248	*0.55*	1.90
**Average**	184.2	97.70%	9.51%	171.4	392.3	46.8	361	97.44%	5081098	4970033	26618	4106636	880928	0.56	1.96
**Minimum**	131.2	73.90%	2.60%	129.5	332.2	38.1	215	95.26%	4763155	4685966	24457	3913210	816697	0.39	1.90
**Maximum**	275.0	99.00%	25.70%	246.6	450.5	62.7	669	98.50%	5853946	5753090	29361	4719925	1033165	0.68	2.04
**Median**	173.3	98.80%	9.50%	166.1	395.7	45.5	321	97.75%	4997891	4879686	26712	4037943	870182	0.565	1.96

DNA was extracted and sequenced using an Illumina HiSeq X with paired 150 nucleotide reads. The average library insert size was 392.3 nt for proband. Sequencing was completed 7 days after consent. rWGS was performed to an average depth of coverage of 47-fold (minimum 38-fold; Table S3). Completeness of rWGS was assessed by the proportion of 15,643 genes in Mendelian Inheritance in Man with ≥10-fold coverage of all coding domain nucleotides (median 97.8%)[1].Rapid alignment and nucleotide variant calling was performed using the Dragen (Edico Genome) hardware and software[2]. Yield was 171.4 Gb for the proband with an average coverage of 46.8x resulting in 4,970,033 distinct variant calls (4,106,636 single nucleotide variants (SNV), 880,928 small insertion deletion variants (indels), 26618 coding domain (CD) variants, with a Ti/Tv ratio of 1.96 (1.90-2.04)). Variants were automatically annotated and analyzed in Opal Clinical (Omicia, Oakland, CA)[3]. Initially, variants were filtered to retain those with allele frequencies of <1% in the Exome Variant Server, 1000 Genomes Samples, and Exome Aggregation Consortium database[4,5]. A differential diagnostic gene list was built in Phenolyzer using the HPO (and SNOMED-CT) codes[6–8]. Yielding a list of potential genes. Variants were further filtered to retain those mapping to the list of potential genes, yielding a small number of variants of interest. Likely pathogenic and pathogenic mutations were curated manually following ACMG criteria[9]. Abbreviations. GB: gigabase; nt: nucleotides; MIM: Mendelian inheritance in Man; CD: coding domain; indels: insertion-deletion nucleotide variants; SNVs: single nucleotide variants; Hom: homozygous; Het: heterozygous; Ti: nucleotide transition; Tv: nucleotide transversion; **Exception pass genome per laboratory director discretion.

Supplementary Table 4: Human Phenotype Ontology (HPO) terms for clinical features, Online Mendelian Inheritance in Man (OMIM)# for diagnoses and type of rWGS test in the twenty-four proband inpatient infants

Patient ID	HPO #	Human Phenotype Ontology Term	Causative Gene	OMIM	rWGS Test
01	HP:0001684	Atrial septal defect		N/A	Solo
	HP:0001331	Absence cavum septum pellucidum, complete			
	HP:0030283	Absence cavum septum pellucidum, partial			
	HP:0007370	Corpus callosum hypoplasia/partial absence			
	HP:0001338	Callosal agenesis or severe hypogenesis			
	HP:0011451	Congenital microcephaly			
	HP:0001276	Hypertonia			
	HP:0006895	Hypertonia, lower limbs			
	HP:0200049	Hypertonia, upper limbs			
	HP:0100490	Campylodactyly			
	HP:0008619	Bilateral hearing loss, sensorineural			
	HP:0008513	Bilateral hearing loss, conductive			
	HP:0000486	Strabismus			
	HP:0000487	Congenital strabismus			
	HP:0004626	Scoliosis			
	HP:0002944	Thoracolumbar scoliosis			
	HP:0004626	Lumbar scoliosis			
	HP:0000921	Missing ribs			
	HP:0001385	Bilateral hip dysplasia, congenital			
	HP:0005407	Low CD4+ T cell subset			
	HP:0001511	In utero growth restriction			
	HP:0000519	Congenital cataracts			
09	HP:0001511	In utero growth restriction		N/A	Solo
	HP:0005160	Total anomalous pulmonary venous return			
	HP:0011682	Perimembranous ventricular septal defect			
	HP:0001684	Moderate atrial septal defect			
	HP:0001171	Ectrodactyly of the right hand			
	HP:0006101	Syndactyly			
	HP:0000308	Microretrognathia			
	HP:0000347	Micrognathia			
	HP:0000219	Thin protruding upper lip			
	HP:0008583	Under folded superior helices			
	HP:0001894	Thrombocytosis			
	HP:0012408	Medullary nephrocalcinosis			
12	HP:0001511	Small for gestational age	*ARID1B*	135900	Solo
	HP:000776	Congenital diaphragmatic hernia			
	HP:0030680	Congenital heart disease			
	HP:0002079	Hypoplasia of corpus callosum			
	HP:0001999	Abnormal face shape			
	HP:0002205	Recurrent respiratory infections			
	HP:0002194	Delayed gross motor development			
18	HP:0001657	Prolonged QT syndrome	*POLR1C*	616494	Trio
	HP:0005184	Prolonged QTc syndrome			
	HP:0002092	Pulmonary hypertension			
	HP:0001385	Hip dysplasia			
	HP:0011451	Congenital microcephaly			
	HP:0011968	Feeding difficulties			
	HP:0002835	Aspiration			
	HP:0001508	Failure to Thrive			
20	HP:0001789	Hydrops fetalis	*TPM1*	611878	Solo
	HP:0001791	Foetal ascites			
	HP:0001698	Pericardial effusion			
	HP:0001711	Left ventricular abnormality			
	HP:0030682	Left ventricular noncompaction			
	HP:0001646	Abnormal aortic valve			
	HP:0012304	Hypoplastic aortic arch			

	HP:0011649	Patent ductus arteriosus after premature birth			
	HP:0001708	Right ventricular failure			
	HP:0009937	Facial hirsutism			
	HP:0000494	Down slanting palpebral fissures			
	HP:0011247	Prominent antihelix, crimped superiorly			
	HP:0010946	Dilation of the renal pelvis			
	HP:0004719	Hyperechogenic kidneys			
	HP:0001873	Thrombocytopenia			
	HP:0001942	Metabolic acidosis			
24	HP:0001680	Coarctation of the aorta	*PHEX*	307800	Trio
	HP:0012304	Hypoplastic aortic arch			
	HP:0011682	Peri-membranous VSD			
	HP:0011670	Left superior vena cava to coronary sinus			
	HP:0011668	Bilateral superior vena cava			
	HP:0001655	Patent foramen ovale			
	HP:0000062	Ambiguous external genitalia at birth			
	HP:0000033	Ambiguous genitalia, male			
	HP:0000808	Penoscrotal hypospadias			
	HP:0000041	Chordee			
	HP:0000960	Sacral dimple			
	HP:0003468	Abnormality of the vertebrae			
	HP:0006380	Knee flexion contractures			
	HP:0001511	In utero growth restriction			
	HP:0000347	Mild micrognathia			
	HP:0001873	Thrombocytopenia			
	HP:0001905	Congenital thrombocytopenia			
26	HP:0002908	Conjugated hyperbilirubinemia	*JAG1 (3Mb deletion)*	118450	Trio
	HP:0001396	Cholestasis			
	HP:0004971	Pulmonary artery hypoplasia			
	HP:0001643	Patent ductus arteriosus			
	HP:0001942	Metabolic acidosis			
	HP:0001947	Renal tubular acidosis			
	HP:0001508	Failure to thrive			
30	HP:0030680	Congenital heart disease	*NF1*	162200	Trio
	HP:0004935	Pulmonary valve atresia	*MYBPC3*	615396	
	HP:0000967	Petechiae			
	HP:0000938	Osteopenia			
	HP:0002788	Recurrent upper respiratory infections			
	HP:0002742	Recurrent Klebsiella infections			
	HP:0002726	Recurrent Staphylococcus infections			
38	HP:0011863	Sternal ossification center anomalies		N/A	Trio
	HP:0001539	Omphalocele			
	HP:0011623	Moderate/large mid muscular VSD			
	HP:0001684	Moderate/large secundum atrial septal defect			
	HP:0004927	Dilated main pulmonary artery			
	HP:0000776	Left posterior congenital diaphragmatic hernia			
	HP:0002092	Pulmonary hypertension			
	HP:0002089	Pulmonary hypoplasia			
	HP:0002878	Respiratory failure			
	HP:0001334	Communicating hydrocephalus			
	HP:0007165	Periventricular heterotopia			
	HP:0010946	Renal pelviectasis			
47	HP:0005298	AVC with right ventricle aorta and pulmonary atresia		N/A	Trio
	HP:0011579				
	HP:0004935	Unbalanced AV canal defect			
	HP:0011563	Pulmonary atresia			
	HP:0011670	Ventriculoarterial discordance			
	HP:0011649	Left superior vena cava to coronary sinus			
	HP:0011701	PDA after premature birth			
	HP:0002104	Atrial tachycardia			
	HP:0006528	Apnea			

	HP:0005100	Chronic lung disease		
	HP:0001508	Premature birth following PROM		
	HP:0008872	Failure to thrive		
		Feeding difficulties in infancy		
49	HP:0010882	Pulmonary valve atresia	N/A	Trio
	HP:0011612	Type A interruption of the pulmonary artery		
	HP:0011604	Large aorto-pulmonary window		
	HP:0001643	Large patent ductus arteriosus		
	HP:0001707	Abnormality of the right ventricle		
	HP:0001714	Ventricular hypertrophy		
	HP:0004762	Severe right ventricular hypoplasia		
	HP:0006704	Right ventricular sinusoids		
	HP:0001647	Bicuspid aortic valve		
	HP:0011726	Persistent fetal circulation		
	HP:0008757	Unilateral vocal cord paralysis		
	HP:0012821	Unilateral vocal cord paresis		
	HP:0001601	Laryngomalacia with redundant arytenoids prolapsing into airway		
	HP:0002625	Deep venous thrombosis		
	HP:0001511	In utero growth restriction		
	HP:0001508	Poor weight gain		
	HP:0002718	Multiple bacterial infections		
	HP:0005420	Multiple bacterial infections with gram negative organisms		
	HP:0000260	Large anterior fontanel		
	HP:0005556	Delayed metopic suture closure		
	HP:0005280	Very depressed nasal bridge		
	HP:0002056	Furrows in glabellar region with flat vascular malformation		
	HP:0001076	Glabellar capillary hemangioma		
	HP:0000463	Anteverted nares		
64	HP:0030853	Heterotaxy syndrome	N/A	Duo
	HP:0001748	Polysplenia		
	HP:0011578	Atrioventricular canal, transitional		
	HP:0010445	Large primum atrial septal defect		
	HP:0001680	Coarctation		
	HP:0001659	Mild aortic valve regurgitation		
	HP:0011103	Abnormality of the left ventricular outflow tract		
	HP:0011589	Left aortic arch with common brachiocephalic		
	HP:0011671	trunk		
		Interrupted inferior vena cava with azygous		
	HP:0011669	connection to the right superior vena cava		
	HP:0004794	Let superior vena cava directly joins left atrium		
	HP:0000121	Small bowel malrotation		
		Nephrocalcinosis		
65	HP:0001680	Severe coarctation of the aorta	N/A	Duo
	HP:0011622	Large inlet ventricular septal defect		
	HP:0011625	Multiple muscular ventricular septal defects		
	HP:0001643	Large patent ductus arteriosus		
	HP:0011667	Bilateral superior vena cava with small bridging		
	HP:0001274	vein		
	HP:0012762	Agenesis of the corpus callosum		
	HP:0030048	Cerebral white matter atrophy		
	HP:0002418	Colpocephaly		
	HP:0001321	Abnormality of midbrain morphology		
	HP:0001320	Hypoplastic cerebellar hemispheres		
	HP:0006951	Hypoplastic cerebellar vermis		
	HP:0000175	Prominent retrocerebellar cyst		
	HP:0000126	Cleft palate		
	HP:0012435	Hydronephrosis		
		Ventral shortening of foreskin		

80	HP:0001674	Complete, balanced atrioventricular canal			Trio
	HP:0001635	Congestive heart failure			
	HP:0009775	Amniotic band syndrome			
	HP:0001873	Thrombocytopenia			
	HP:0002580	Volvulus			
	HP:0005214	Small bowel obstruction			
	HP:0100584	Endocarditis			
	HP:0004887	Respiratory failure, post-operative			
82	HP:0005164	Dysplastic pulmonary valve	*KMT2D*	147920	Quad
	HP:0001642	Pulmonary valve stenosis			
	HP:0030732	Dysplastic tricuspid valve			
	HP:0001667	Right ventricular hypertrophy			
	HP:0011648	Patent ductus arteriosus after term birth			
	HP:0000470	Short neck			
	HP:0005989	redundant skin in nuchal area			
	HP:0000278	Mild retrognathia			
	HP:0001873	Thrombocytopenia			
	HP:0000836	Hyperthyroidism			
		Neonatal Graves' Disease			
84	HP:0001719	Double outlet right ventricle		N/A	Trio
	HP:0001629	Large VSD			
	HP:0001643	Large PDA			
		Shone's complex			
	HP:0012304	Hypoplastic aortic arch			
	HP:0011611	Transverse arch hypoplasia			
	HP:0001680	Focal coarctation			
		Dysplastic/hypoplastic mitral valve			
		Mild LV hypoplasia			
	HP:0001647	Bicuspid aortic valve			
	HP:0000400	Large ears with uplifted lobes			
	HP:0000448	Prominent, large nose			
	HP:0001176	Large hands			
	HP:0001833	Large feet			
	HP:0001541	Ascites			
	HP:0002202	Pleural effusions			
	HP:0001688	Sinus Bradycardia			
		Hypoxic Ischemic Encephalopathy			
	HP:0002637	Brain ischemia			
	HP:0001250	Seizures			
	HP:0003128	Lactic Acidosis			
86		VACTERL association			Trio
	HP:0001627	Congenital heart defects			
	HP:0001629	Moderate-large VSD			
	HP:0002623	Aortic valve override			
	HP:0001684	Moderate secundum ASD			
	HP:0001642	Mild pulmonary stenosis			
	HP:0011670	Left SVC to coronary sinus			
	HP:0002575	Tracheoesophageal fistula			
	HP:0002032	Esophageal atresia			
	HP:0002023	Imperforate Anus			
	HP:0010479	Patent Urachus			
	HP:0000076	Vesicoureteral reflux			
		grade 5 bilaterally			
		Duplicated urethra			
	HP:0000795	Abnormality of urethra			

	HP:0000028	Undescended testes			
	HP:0003316	Butterfly Vertebrae			
		Limb anomalies			
	HP:0001776	Club feet			
	HP:0003974	Right upper extremity missing radial bone			
	HP:0000843	Elevated Parathyroid hormone			
	HP:0000083	Renal failure			
	HP:0000822	Hypertension			
	HP:0009894	Abnormal ears, thickened			
92	HP:0010775	Vascular Ring of Aorta	*CHD7*	214800	Trio
	HP:0010296	Ankyloglossia			
	HP:0002835	Aspiration, Silent Aspiration			
	HP:0001601	Laryngomalacia			
	HP:0001607	Subglottic stenosis			
	HP:0000126	Pelviectasis , hydronephrosis			
96	HP:0011726	Persistent Pulmonary Hypertension of the	*FOXF1*	265380	Solo
	HP:0010773	Newborn (PPHN), Persistent Fetal Circulation			
		Partial Anomalous Pulmonary Venous Return			
100	HP:0002251	Aganglionic megacolon	*ZEB2*	235730	Trio
	HP:0001290	Generalized Hypotonia			
	HP:0008743	Coronal Hypospadias			
	HP:0001273	Abnormality of corpus callosum			
	HP:0001642	Pulmonic stenosis			
	HP:0001629	Ventricular septal defect			
	HP:0000288	Abnormality of philtrum			
104	HP:0001642	Pulmonary Stenosis		N/A	Trio
	HP:0010773	Partial anomalous pulmonary venous return to			
	HP:0011625	SVC			
	HP:0011664	Ventricular septal defects, multiple			
	HP:0001716	Left Ventricular Non-compaction			
	HP:0010296	cardiomyopathy			
		Wolff-Parkinson-White			
		Ankyloglossia			
108	HP:0009729	Cardiac Rhabdomyoma	*TSC2*	613254	Trio
	HP:0010568	Hamartoma of the eye			
	HP:0009731	Cerebral hamartomas			
112	HP:0001511	In Utero Growth Restriction			Trio
	HP:0000776	Congenital Diaphragmatic Hernia			
	HP:0002575	Tracheoesophageal Fistula			
	HP:0001561	Polyhydramnios			
	HP:0008386	Hypoplastic nail beds			
	HP:0000122	Single kidney (unilateral renal agenesis)			
	HP:0012304	Aortic arch hypoplasia			
	HP:0000175	Cleft palate			
	HP:0008551	Microtia			
	HP:0004209	5th Finger Clinodactyly			
	HP:0002119	Ventriculomegaly			
		Wide spaced nipples			
114	HP:0000776	Congenital diaphragmatic hernia		N/A	Trio
	HP:0011611	Interrupted aortic arch			
	HP:0001269	Ventricular septal defect			
	HP:0004383	Hypoplastic left heart			
	HP:0002092	Pulmonary Hypertension			
		Mitral valve hypoplasia			

Supplementary Table 5: Cardiac defects, rWGS and Clinical Genetic tests metrics

ID	Cardiac Dx	Age at Consent (days)	rWGS Test	rWGS result	Turn Around Time	Diagnosis	Microarray	Targeted Gene Panel
01	Moderate atrial septal defect (ASD)	120	Solo	N	4	N/A	Normal	
09	Total anomalous pulmonary venous return, Peri-membranous ventricular septal defect (VSD), Moderate ASD, Patent Ductus Arteriosus (PDA)	145	Solo	N	22	N/A	Normal	
12	Shone's Complex, Bicuspid aortic valve, Coarctation, Peri-membranous VSD, PDA	231	Solo	LP	15	Coffin-Siris Syndrome (OMIM# 135900)	Normal	
18	Bicuspid Aortic valve, ASD, Cardiomegaly	29	Trio	LP	31	leukodystrophy, hypomyelinating (OMIM# 616494)	Normal	Long QT Panel: negative (AKAP9, ANK2, CACNA1C, CALM1, CALM3, CAV3, KCNE1, KCNE2, KCNH2, KCNJ2, KCNJ5, KCNQ1, SCN4B, SCN5A, SNTA1, TRDN)
20	Non-apex forming left ventricle (LV), Suspect LV Non-Compaction, PDA, Cardiac Failure	5	Solo	LP	26	Left Ventricular Noncompaction Syndrome (OMIM# 611878)	N/A	Rasopathy Panel: negative (A2ML1, BRAF, HRAS, KRAS, MAP2K1, MAP2K2, NF1, NRAS, PTPN11, RAF1, RASA1, RIT1, SHOC2, SOS1, SPRED1)
24	Coarctation of the aorta, transverse arch hypoplasia, Perimembranous VSD, Left Superior Vena Cava (LSVC) to Coronary Sinus (CS), Congestive heart failure	137	Trio	LP	14	X-linked Hypophosphatemic Rickets Syndrome (OMIM# 307800)	Normal	
26	Significant LPA hypoplasia with proximal stenosis. Mild RPA hypoplasia. Tortuous PDA. Aberrant Right subclavian artery.	80	Trio	P	5	Alagille Syndrome (OMIM# 118450)	Pathogenic: 3.0Mb deletion of 20p12.2p12.1-Alagille Syndrome	
30	Pulmonary Atresia/Intact Ventricular Septum (PA/IVS) with coronary sinusoids communicating to	227	Trio	LP/ LP	13	Neurofibromatosis Type 1 OMIM# 162200) Cardiomyopathy	Normal	

	bilateral coronary arterial vasculature					(OMIM# 615396)		
38	Pentalogy of Cantrell: Large mid-muscular VSD, Moderate ASD, LSVC to CS, Aberrant Right Subclavian	131	Trio	N	21	N/A	Normal	
47	Unbalanced Atrioventricular canal (AVC), PA, ventriculoarterial discordance	58	Trio	N	17	N/A	Normal	
49	PA/IVS, Interrupted Aortic Arch, Bicuspid Aortic Valve	168	Trio	N	34	N/A	Normal	
64	Heterotaxy Syndrome, AVC, Coarctation	83	Duo	N	14	N/A	Normal	
65	Coarctation of the Aorta, Multiple VSDs, Bilateral SVCs	16	Duo	N	20	N/A	Normal	
80	Complete balanced AVC, Congestive Heart Failure	125	Trio	N	15	N/A	Normal	
82	Pulmonary valve stenosis, dysplastic pulmonary valve, Right Ventricular hypertrophy	15	Quad	LP	11	Kabuki Syndrome (OMIM# 147920)	Normal	
84	Double Outlet Right Ventricle, Shone's Complex, large VSD	22	Trio	N	29	N/A	VUS: 31Kb deletion 9p24.3	
86	Large VSD, Overriding Aorta, LSVC to CS	54	Trio	N	15	N/A	Normal	
92	Vascular Ring: right sided Aortic Arch with anomalous Left Subclavian Artery, Mildly hypoplastic/bicuspid Aortic Valve, Abnormal Mitral valve, Moderate mitral stenosis	59	Trio	P	10	CHARGE Syndrome (OMIM# 214800)	N/A	
96	Partial Anomalous Pulmonary Venous Return (PAPVR), Right atrial enlargement. Severe right ventricular hypertension. Left aortic arch with common brachiocephalic trunk. LPA borderline hypoplastic.	37	Solo	LP	10	Alveolar Capillary Dysplasia with misalignment of pulmonary veins (Dx 5/18) (OMIM# 265380)	N/A	Surfactant Panel: VUS FOXF1 c.188G>T p.Ser63Ile, Het, AD.
100	Small anterior muscular and tiny mid muscular type VSD. Doming and thickened pulmonic valve with moderate to severe pulmonary stenosis, PDA	9	Trio	P	8	Mowat-Wilson Syndrome (OMIM# 235730)	Normal	
104	Swiss cheese apical muscular VSDs. PAPVR to SVC. Thickened and doming pulmonic valve.	79	Trio	N	12	N/A	N/A	

	Moderate RA dilation, mildly dilated left atrium. Papillary muscles slightly asymmetric. Posterior leaflet of the mitral valve is shorter. LV appears non-compacted and mildly hypertrophied. Diminished LV function.						
108	Multiple rhabdomyomas. Very large mass associated with the RV free wall. Multiple small masses in the LV free wall and near the anterolateral papillary muscle. Large PDA	3	Trio	P	13	Tuberous Sclerosis (OMIM# 613254)	N/A
112	Hypoplastic transverse arch with concern for critical arch obstruction, mild hypoplasia of the ascending aorta. Large PDA, LPA hypoplasia. Rightward displaced heart.	1	Trio	N	26	N/A	Normal
114	Interrupted aortic arch type B with aberrant right subclavian, moderate-large cono-ventricular type VSD. Moderately hypoplastic mitral valve. Hypoplastic aortic valve, bicuspid. Moderate hypoplasia of the ascending aorta. Large PDA, LPA is mildly hypoplastic.	6	Trio	N	12	N/A	Normal

Probands age of consent ranged from DOL1 to DOL23. There was varied cardiac diagnosis ranging from moderate atrial septal defect to more complex diagnosis like Pulmonary Atresia Intact Ventricular septum. Sixteen of the 24 families (67%) received trio sequencing (proband and parents), 5 (21%) solo (proband only), 2 (8%) duo (proband and mother), and 1 (4%) quad (proband, parents and an affected sibling). All but one had only 1 affected family member (the proband). The family that underwent quad rWGS had two affected children and one affected parent. Twenty-one of 24 probands had clinical genetic testing in the form of microarray and/or targeted gene panels. Three children had no additional genetic testing. Molecular diagnoses ranged from syndromes affecting multiple organ systems to disorders limited to the cardiovascular system. ASD=Atrial Septal Defect, VSD=Ventricular Septal Defect, PDA=Patent Ductus Arteriosus, LV= Left Ventricle, LSVC= Left Superior Vena Cava, CS= Coronary Sinus, LPA=Left Pulmonary Artery, RPA=Right Pulmonary Artery, PA/IVS=Pulmonary Atresia/Intact Ventricular Septum, AVC=Atrioventricular Canal, PA=Pulmonary Atresia, SVC=Superior Vena Cava, PAPVR= Partial Anomalous Pulmonary Venous Return, OMIM= Online Mendelian Inheritance in Man, AKAP9=A-kinase anchor protein 9, ANK2=Ankyrin 2, CACNA1C=Calcium Voltage-Gated Channel Subunit Alpha1 C, CALM1=Calmodulin 1, CALM3=Calmodulin 3, CAV3=Caveolin-3, KCNE1=Potassium Voltage-Gated Channel Subfamily E member 1, KCNE2= Potassium Voltage-Gated Channel Subfamily E member 2, KCNH2= Potassium Voltage-Gated Channel Subfamily H Member 2, KCNJ2= Potassium Inwardly Rectifying Channel Subfamily J Member 2, KCNJ5= Potassium Inwardly Rectifying Channel Subfamily J Member 5, KCNQ1= Potassium Voltage-Gated Channel Subfamily Q Member 1, SCN4B= Sodium channel β-subunit 4, SCN5A= Sodium Voltage-Gated Channel Alpha Subunit 5, SNTA1= Syntrophin Alpha 1, TRDN=Triadin, A2ML1= alpha-2-macroglobulin like 1, BRAF= B-Raf Proto-Oncogene, Serine/Threonine Kinase, HRAS= HRas Proto-Oncogene, GTPase, KRAS= KRAS Proto-Oncogene, MAP2K1= Dual specificity mitogen-activated protein kinase 1, MAP2K2= Dual specificity mitogen-activated protein kinase 2, NF1= Neurofibromatosis type 1, NRAS= NRas Proto-Oncogene, PTPN11= Protein Tyrosine Phosphatase Non-Receptor Type 11, RAF1= Raf-1 Proto-Oncogene, Serine/Threonine Kinase, RASA1= RAS p21 protein activator 1, RIT1= Ras-Like Without CAAX Protein 1, SHOC2= Leucine-Rich Repeat Protein SHOC-2, SOS1= Son of sevenless homolog 1, SPRED1= Sprouty Related EVH1 Domain Containing 1, FOXF1= Forkhead Box F1, N=Negative, P=Pathogenic, LP=Likely Pathogenic, N/A= Not Applicable, VUS= Variant of Unknown Significance.

Supplementary Table 6: Neurodevelopmental/ Infectious/ Immunological and Endocrinological implications of rWGS diagnosis

Patient ID	Disease Associations	Effect on Management	Neurodevelopmental Associations	Infectious/Immunological/Endocrinological Associations
12	Coffin-Siris Syndrome (OMIM# 135900)	Palliative Care	Feeding problems (90%), Hypotonia (75%), Seizures (50%) Hearing impairment (45%),Visual Impairment (~40%), Developmental delay, Intellectual disability, Behavioral abnormalities	Frequent Infections, mostly upper respiratory infections
18	Hypomyelinating leukodystrophy (OMIM# 616494)	Enlistment of additional subspecialists	Delayed psychomotor development, loss/lack of independent ambulation, abnormal cognition, tremor, ataxia, spasticity, cerebellar signs	Hypogonadotropic hypogonadism
20	Left Ventricular (LV) Noncompaction (OMIM# 611878)	Listing for Cardiac Transplantation		
24	X-linked hereditary hypophosphatemic rickets (XLHR) (OMIM# 307800)	Enlistment of additional subspecialists	Deafness appearing in adulthood (rare)	Reduction in renal phosphate reabsorption per glomerular filtration rate to ~50% of normal
26	Alagille Syndrome (OMIM# 118450)	Avoidance of Intraoperative Cholangiogram	Absent deep tendon reflexes, poor school performance	Growth retardation unresponsive to growth hormone therapy (may benefit from IGF1 treatment instead)
30	Neurofibromatosis (OMIM# 162200)	Enlistment of additional subspecialists	Macrocephaly/ short stature, Vision loss due to optic gliomas in ~47% of patients by 4 year of age, Cognitive deficits in language and reading abilities, impaired visual spatial and neuromotor skills	
	Hypertrophic and Dilated Cardiomyopathy, LV Noncompaction (OMIM# 615396)	Medication Change		
82	Kabuki Syndrome (OMIM# 147920)	Enlistment of additional subspecialists	Mild to moderate mental retardation, Post-natal growth deficiency	Recurrent otitis media, congenital hypothyroidism, Hypogammaglobulinemia (low IgA, IgG)
92	CHARGE syndrome (OMIM# 214800)	Enlistment of additional subspecialists	Developmental delay, growth retardation	T cell abnormalities

96	Alveolar Capillary Dysplasia with misalignment of Pulmonary Veins (OMIM# 265380)	Avoidance of lung biopsy. Transfer to Pulmonary transplant center	
100	Mowat-Wilson Syndrome (OMIM# 235730)	Enlistment of additional subspecialists	Mental retardation, microcephaly (sometimes), short stature, epilepsy
108	Tuberous Sclerosis type 2 (OMIM# 613254)	Targeted Genetic Counseling (TSC2 more severe phenotype than TSC1)	Epilepsy, learning difficulties, behavioral problems

rWGS diagnosis had implications for neurodevelopment in 82% (9/11) of the probands and had endocrinologic, immunologic and/or infectious concerns that could impact short and long-term outcome in 52% (6/11). LV= Left Ventricle, IGF1= Insulin Like Growth Factor 1, IgA=Immunoglobulin A, IgG=Immunoglobulin G, TSC2=Tuberous Sclerosis 2, TSC1=Tuberous Sclerosis 1, OMIM= Online Mendelian Inheritance in Man.

Supplementary Table 7. Cardiac defects, surgical interventions/procedures, Costs trends around rWGS

ID	Cardiac Dx	Age at Consent (days)	rWGS results		DOA-DOBC		DOBC to DOR		DOR to DODC	
01	Moderate atrial septal defect (ASD)	120	N	Avg Daily Hospital Cost ($)	5,347.29		3,206.00		639.00	
					Surgical Interventions<br>Laparoscopic fundoplication with gastrostomy<br>None	Intubations/ Procedures<br>Skin Biopsy	Surgical Interventions	Intubations/ Procedures	Surgical Interventions	Intubations/ Procedures
09	Total anomalous pulmonary venous return, Peri-membranous ventricular septal defect (VSD), Moderate ASD, Patent Ductus Arteriosus (PDA)	145	N	Avg Daily Hospital Cost ($)	5,221.47		9,693.96		4,242.34	
					Surgical Interventions	Intubations/ Procedures	Surgical Interventions<br>Repair TAPVR, VSD closure, removal PA band and patch reconstruction of the MPA	Intubations/ Procedures	Surgical Interventions<br>Fundoplication with gastrostomy	Intubations/ Procedures
12	Shone's Complex, Bicuspid aortic valve, Coarctation, Peri-membranous VSD, PDA	231	LP	Avg Daily Hospital Cost ($)	7,103.56		8,982.15		5,067.00	
					Surgical Interventions<br>Arch repair with patch homograft, VSD/ASD closure<br><br>ECMO<br>delayed sternal closure<br>G-tube Nissen	Intubations/ Procedures<br>Bronchoscopy and direct laryngoscopy	Surgical Interventions	Intubations/ Procedures	Surgical Interventions	Intubations/ Procedures<br>Oscillatory Ventilation

					Exploratory Laparotomy<br><br>Tracheostomy					
18	Bicuspid Aortic valve, ASD, Cardiomegaly	29	LP	Avg Daily Hospital Cost ($)	8,602.00		4,649.96		4,132.00	
					Surgical Interventions<br>Left thoracotomy for temporary pacing wires	Intubations/ Procedures	Surgical Interventions<br>Pacemaker implantation	Intubations/ Procedures	Surgical Interventions	Intubations/ Procedures
20	Non-apex forming left ventricle (LV), Suspect LV Non-Compaction, PDA, Cardiac Failure	5	LP	Avg Daily Hospital Cost ($)	13,862.00		9,777.00		7,721.00	
					Surgical Interventions	Intubations/ Procedures	Surgical Interventions<br>Bilateral PA Bands<br><br>Delayed Sternal Closure<br><br>PDA stent	Intubations/ Procedures<br>Brain MRI	Surgical Interventions<br>Orthotopic Heart Transplant<br><br>Fundoplication and G-tube	Intubations/ Procedures
24	Coarctation of the aorta, transverse arch hypoplasia, Peri-membranous VSD, Left Superior Vena Cava (LSVC) to Coronary Sinus (CS), Congestive heart failure	137	LP	Avg Daily Hospital Cost ($)	6,466.49		7,559.14		5,017.24	
					Surgical Interventions<br>PA banding<br><br>DSC<br>Repair aortic arch, VSD, ASD<br><br>DSC<br><br>L diaphragm plication	Intubations/ Procedures<br>Direct laryngo-bronchoscopy<br><br>MRI-Spine	Surgical Interventions	Intubations/ Procedures	Surgical Interventions	Intubations/ Procedures

					G-tube<br>Fundoplication					
26	Significant LPA hypoplasia with proximal stenosis. Mild RPA hypoplasia. Tortuous PDA. Aberrant Right subclavian artery.	80	P	Avg Daily Hospital Cost ($)	2,344.00		4,115.00		2,402.00	
					Surgical Interventions	Intubations/ Procedures<br>Fluoroscopy<br><br>Cardiac CT	Surgical Interventions	Intubations/ Procedures	Surgical Interventions	Intubations/ Procedures
30	Pulmonary Atresia/Intact Ventricular Septum (PA/IVS) with coronary sinusoids communicating to bilateral coronary arterial vasculature	227	LP/ LP	Avg Daily Hospital Cost ($)	7,673.37		7,379.23		7,075.50	
					Surgical Interventions<br>Atrial septectomy, PDA ligation, Central AP shunt<br><br>DSC<br><br>Bilateral PA banding<br><br>Tracheostomy<br><br>G-tube/Fundo	Intubations/ Procedures<br>DLB	Surgical Interventions	Intubations/ Procedures	Surgical Interventions<br>Hickman Catheter insertion	Intubations/ Procedures
38	Pentalogy of Cantrell: Large mid-muscular VSD, Moderate ASD, LSVC to CS, Aberrant Right Subclavian	131	N	Avg Daily Hospital Cost ($)	6,930.00		7,437.52		5,689.00	
					Surgical Interventions<br>left diaphragmatic hernia repair<br><br>Diaphragm revision<br><br>Tracheostomy	Intubations/ Procedures<br>Laryn-goscopy/ bronchospy<br><br>Cardiac Cath	Surgical Interventions<br>VP Shunt	Intubations/ Procedures	Surgical Interventions	Intubations/ Procedures

					Circumcision<br><br>Etv (endoscopic third ventriculostomy)					
47	Unbalanced Atrioventricular canal (AVC), PA, ventriculoarterial discordance	58	N	Avg Daily Hospital Cost ($)	6,459.00		6,655.00		7,009.00	
					Surgical Interventions	Intubations/ Procedures Re-intubation	Surgical Interventions	Intubations/ Procedures Fluoroscopy	Surgical Interventions PDA ligation, bidirectional shunt placement and LPA plasty<br><br>DSC	Intubations/ Procedures
49	PA/IVS, Interrupted Aortic Arch, Bicuspid Aortic Valve	168	N	Avg Daily Hospital Cost ($)	8,044.05		9,586.00		6,863.97	
					Surgical Interventions	Intubations/ Procedures	Surgical Interventions reconstruction of ascending Aorta/ Aortoplasty, Bilateral PA plasty, BT shunt removal, central AP Shunt (3.5mm)<br><br>Delayed Sternal Closure	Intubations/ Procedures	Surgical Interventions	Intubations/ Procedures
64	Heterotaxy Syndrome, AVC, Coarctation	83	N	Avg Daily Hospital Cost ($)	5,198.00		10,084.00		5,721.00	
					Surgical Interventions	Intubations/ Procedures	Surgical Interventions Transitional AV canal	Intubations/ Procedures	Surgical Interventions	Intubations/ Procedures

							repair, LSVC baffle to RA, Closure primum ASD<br><br>Delayed Sternal Closure			
65	Coarctation of the Aorta, Multiple VSDs, Bilateral SVCs	16	N	Avg Daily Hospital Cost ($)	N/A		N/A		N/A	
					Surgical Interventions<br><br>PA Band	Intubations/ Procedures	Surgical Interventions<br><br>DSC	Intubations/ Procedures	Surgical Interventions	Intubations/ Procedures
80	Complete balanced AVC, Congestive Heart Failure	125	N	Avg Daily Hospital Cost ($)	9,789.00		6,983.00		3,032.00	
					Surgical Interventions<br>Complete AV canal repair	Intubations/ Procedures	Surgical Interventions	Intubations/ Procedures	Surgical Interventions	Intubations/ Procedures
82	Pulmonary valve stenosis, dysplastic pulmonary valve, Right Ventricular hypertrophy	15	LP	Avg Daily Hospital Cost ($)	N/A		N/A		N/A	
					Surgical Interventions	Intubations/ Procedures<br>Pulmonary Balloon Valvuloplasty	Surgical Interventions	Intubations/ Procedures	Surgical Interventions	Intubations/ Procedures
84	Double Outlet Right Ventricle, Shone's Complex, large VSD	22	N	Avg Daily Hospital Cost ($)	12,294.00		8,813.00		7,795.00	
					Surgical Interventions	Intubations/ Procedures	Surgical Interventions<br>PD drain<br><br>Exploratory laparotomy, ileostomy<br>Decreased	Intubations/ Procedures<br>MRI<br><br>Abdominal CT	Surgical Interventions	Intubations/ Procedures
86	Large VSD, Overriding	54	N	Avg Daily Hospital Cost ($)	4,726.00		6,867.00		6,512.00	

	Aorta, LSVC to CS				Surgical Interventions	Intubations/ Procedures	Surgical Interventions PD drain placement and excision of umbilical polyp/ granuloma Increased	Intubations/ Procedures	Surgical Interventions Repair VSD	Intubations/ Procedures
92	Vascular Ring: right sided Aortic Arch with anomalous Left Subclavian Artery, Mildly hypoplastic/bicuspid Aortic Valve, Abnormal Mitral valve, Moderate mitral stenosis	59	P	Avg Daily Hospital Cost ($)	5,336.00		6,003.20		4,512.00	
					Surgical Interventions	Intubations/ Procedures Re-intubations X2	Surgical Interventions Laryngo-tracheal reconstruction<br><br>Vascular Ring division	Intubations/ Procedures	Surgical Interventions DLB and Supraglotto-plasty	Intubations/ Procedures Re-intubation
96	Partial Anomalous Pulmonary Venous Return (PAPVR), Right atrial enlargement. Severe right ventricular hypertension. Left aortic arch with common brachiocephalic trunk. LPA borderline hypoplastic.	37	LP	Avg Daily Hospital Cost ($)	8,875.00		4,756.30		5,217.12	
					Surgical Interventions	Intubations/ Procedures	Surgical Interventions	Intubations/ Procedures	Surgical Interventions	Intubations/ Procedures
100	Small anterior muscular and tiny mid muscular type VSD. Doming and thickened pulmonic valve with moderate to	9	P	Avg Daily Hospital Cost ($)	5,017.00		4,611.00		6,134.00	
					Surgical Interventions	Intubations/ Procedures	Surgical Interventions	Intubations/ Procedures Rectal suction Biopsy	Surgical Interventions laparoscopic SOAVE pull-	Intubations/ Procedures

	severe pulmonary stenosis, PDA							PICC line	through surgery	
104	Swiss cheese apical muscular VSDs. PAPVR to SVC. Thickened and doming pulmonic valve. Moderate RA dilation, mildly dilated left atrium. Papillary muscles slightly asymmetric. Posterior leaflet of the mitral valve is shorter. LV appears non-compacted and mildly hypertrophied. Diminished LV function.	79	N	Avg Daily Hospital Cost ($)	5,343.00		3,522.00		294.00	
					Surgical Interventions	Intubations/ Procedures	Surgical Interventions	Intubations/ Procedures	Surgical Interventions	Intubations/ Procedures
108	Multiple rhabdomyomas. Very large mass associated with the RV free wall. Multiple small masses in the LV free wall and near the anterolateral papillary muscle. Large PDA	3	P	Avg Daily Hospital Cost ($)	N/A		N/A		N/A	
					Surgical Interventions	Intubations/ Procedures Brain MRI	Surgical Interventions	Intubations/ Procedures	Surgical Interventions	Intubations/ Procedures
112	Hypoplastic transverse arch with concern for critical arch obstruction, mild hypoplasia of the ascending aorta. Large	1	N	Avg Daily Hospital Cost ($)	N/A		N/A		N/A	
					Surgical Interventions	Intubations/ Procedures	Surgical Interventions	Intubations/ Procedures	Surgical Interventions	Intubations/ Procedures

	PDA, LPA hypoplasia.									
114	Rightward displaced heart. Interrupted aortic arch type B with aberrant right subclavian, moderate-large cono-ventricular type VSD. Moderately hypoplastic mitral valve. Hypoplastic aortic valve, bicuspid. Moderate hypoplasia of the ascending aorta. Large PDA, LPA is mildly hypoplastic.	6	N	Avg Daily Hospital Cost ($)	12,626.00		9,591.00		6,855.00	
					Surgical Interventions<br>Congenital Diaphragmatic Hernia repair	Intubations/ Procedures	Surgical Interventions	Intubations/ Procedures	Surgical Interventions<br>Repair IAA, VSD<br>DSC<br>LADDs/Nissen Gastrostomy tube	Intubations/ Procedures

Spending in period 2 (DOBC to DOR) was dichotomous: 50% (n=10) of the cohort had increased cost relative to period 1, while 50% (n=10) had decreased cost. One explanation for this finding is the higher frequency of major surgical procedures, cardiac and other, in patients with increased cost during this period (60%) compared with the group with decreased cost (30%); however, this difference did not reach statistical significance likely due to sample size. There was no difference in the number of patients who underwent expected cardiac surgical procedures in the two groups. ASD=Atrial Septal Defect, VSD=Ventricular Septal Defect, PDA=Patent Ductus Arteriosus, LV= Left Ventricle, LSVC= Left Superior Vena Cava, CS= Coronary Sinus, LPA=Left Pulmonary Artery, RPA=Right Pulmonary Artery, PA/IVS=Pulmonary Atresia/Intact Ventricular Septum, AVC=Atrioventricular Canal, PA=Pulmonary Atresia, SVC=Superior Vena Cava, PAPVR= Partial Anomalous Pulmonary Venous Return, MPA=Main Pulmonary Artery, ECMO=Extracorporeal Membrane Oxygenation, OHT=Orthotopic Heart Transplant, DSC=Delayed Sternal Closure, AP=Aortopulmonary Shunt, BT=Blalock-Taussig, AV=Atrioventricular, IAA=Interrupted Aortic Arch, DLB: Direct Laryngo-bronchoscopy N=Negative, P=Pathogenic, LP=Likely Pathogenic, N/A= Not Applicable, Avg=Average.

Supplementary References

1. Amberger JS, Bocchini CA, Schiettecatte F, Scott AF, Hamosh A. OMIM.org: Online Mendelian Inheritance in Man (OMIM®), an Online catalog of human genes and genetic disorders. *Nucleic Acids Res*. 2015. doi:10.1093/nar/gku1205
2. Miller NA, Farrow EG, Gibson M, et al. A 26-hour system of highly sensitive whole genome sequencing for emergency management of genetic diseases. *Genome Med*. 2015. doi:10.1186/s13073-015-0221-8
3. Coonrod EM, Margraf RL, Russell A, Voelkerding K V., Reese MG. Clinical analysis of genome next-generation sequencing data using the Omicia platform. *Expert Rev Mol Diagn*. 2013. doi:10.1586/14737159.2013.811907
4. EVS. Exome Variant Server. *NHLBI GO Exome Seq Proj*. 2014.
5. Karczewski KJ, Weisburd B, Thomas B, et al. The ExAC browser: Displaying reference data information from over 60 000 exomes. *Nucleic Acids Res*. 2017. doi:10.1093/nar/gkw971
6. Yang H, Robinson PN, Wang K. Phenolyzer: Phenotype-based prioritization of candidate genes for human diseases. *Nat Methods*. 2015. doi:10.1038/nmeth.3484
7. NIH-NLM. SNOMED Clinical Terms® (SNOMED CT®). *NIH-US Natl Libr Med*. 2015.
8. Köhler S, Vasilevsky NA, Engelstad M, et al. The human phenotype ontology in 2017. *Nucleic Acids Res*. 2017. doi:10.1093/nar/gkw1039
9. Richards S, Aziz N, Bale S, et al. Standards and guidelines for the interpretation of sequence variants: A joint consensus recommendation of the American College of Medical Genetics and Genomics and the Association for Molecular Pathology. *Genet Med*. 2015. doi:10.1038/gim.2015.30
